# Census-based adolescent depression risk assessment: A novel method and reflections

**DOI:** 10.1371/journal.pone.0357661

**Published:** 2026-09-15

**Authors:** Qiang Li, Yangxinrong Tang, Yiling Shi, Yufeng Wu, Zhan Xu, Hefeng Zhou, James J. Zhang

**Affiliations:** 1 School of Physical Education, Shangrao Normal University, Shangrao, Jiangxi, China; 2 New York University, Graduate School of Arts and Science, New York, United States of America; 3 Department of Psychosis Studies, Institute of Psychiatry, Psychology and Neuroscience, King’s College London, London, United Kingdom; 4 School of Agriculture and Biology, Shanghai Jiao Tong University, Shanghai, China; 5 University of Michigan Ann Arbor, Ann Arbor, Michigan, United States of America; 6 Department of Informatics, King’s College London, London, United Kingdom; 7 Department of Kinesiology, University of Georgia, Athens, United States of America; Mansoura University Faculty of Medicine, EGYPT

## Abstract

Diagnosing psychological issues in adolescents is generally more challenging than in adults. The need to enhance early identification and risk assessment for adolescent mental health problems has become particularly urgent, especially given the surge in such issues brought about by the COVID-19 pandemic and its isolation policies. Traditional approaches relying on scales or interviews can be difficult to administer at scale and may miss at-risk youths, while recent attempts at artificial intelligence–assisted clinical diagnosis have not yet achieved consistent performance for routine clinical use. Using youth census survey data from the National Survey of Children’s Health (NSCH) from 2020 to 2022 (147,772 samples), we propose a novel data synthesis and training management method tailored for imbalanced health data, and develop a heuristic decision model based on daily-life indicators to predict survey-reported adolescent depression risk. Experimental results demonstrate that our data management approach improves the average accuracy of conventional models by 14%. Compared with general artificial intelligence methods, the proposed decision model achieves higher accuracy, precision, and sensitivity. Built on AutoML, the model can adapt to factor replacement and incremental data updates, supporting scalable screening workflows and model maintenance. Using Shapley values, we identify several key daily-life factors associated with adolescent depression risk, such as social interactions and sleep duration. The interpretability analysis further supports the utility of our approach for risk stratification and practical decision support, rather than replacing clinician-administered diagnosis.

## Introduction

In the face of the fast-paced society, mental illness has increasingly become a contemporary factor affecting individuals’ health, and proportion of people suffering from psychological disorders is constantly increasing [1]. Research has shown that since the 21st century, the mental health of adolescents in developed countries such as the United States, the United Kingdom, and Finland has significantly deteriorated [2–5]. The COVID-19 pandemic has caused regional casualties, and some countries and regions have also implemented varying degrees of lockdowns to resist the epidemic. Though the WHO has announced the end of the pandemic, its impact on the mental health of adolescents still exists, and the mental health issues of adolescents in the post pandemic era have become one of the top mental health issues at present [6,7].

Individuals experience significant social role transitions during adolescence, which is a period of rapid growth. In this period of transition from childhood to adulthood, it is necessary to make academic and professional decisions, and establish a foundational positioning for the future. Along with it comes identity development and academic pressure, which can greatly affect the mental health of adolescents [8]. In addition, due to the immature physical and mental development of adolescents, various unfavorable factors in their growth environment, such as cold parent-child relationships, lack of warm dependence, tense family atmosphere, rough educational methods, and suppressed peer relationships, may lead to problematic behavior among adolescents [9–11]. The psychological problems of children and adolescents rarely attract the attention of general practitioners [12], and adolescent psychological problems are often overlooked by guardians. Therefore, the diagnosis and treatment of adolescent psychological problems are crucial for personal development and even the future situation of families. The earlier psychological problems are discovered and treated, the better the prognosis, while also saving expensive treatment costs for mental illnesses.

Early diagnosis and prediction of diseases are of great significance for improving public health conditions, personal economic expenses, and treatment outcomes [13]. Recently, Computer-Aided Diagnosis (CAD) becomes mature and widely used technology [14–16], but the currently better CAD methods are limited to specific diseases, imaging modalities, or Artificial Intelligence (AI) techniques. Especially in the early prediction and diagnosis of AI, only cardiovascular diseases, diabetes and some cancers can have a better effect [17–21]. Compared to pathological detection and prediction, there are fewer cases involving psychological diagnosis and treatment applications [22]. European and American countries are at the forefront of big data based mental health research, and there is currently a certain amount of related research on mental health analysis and prediction [23–25]. In early stage, the ultra high-risk model was proposed for better identify potential mental problems and provide early intervention and treatment [26]. Researchers used multi-level modeling of internet search patterns to identify risk factors of interest [27]. With the advancement of AI technologies, methods for predicting psychological issues have been significantly improved. A big-data-based approach has demonstrated promising performance in suicide prediction research. [28]. However, in the practical application of handling massive amounts of data [24,29,30], there are still deficiencies. Few achievements have been successfully applied to clinical diagnosis.

The traditional and primary diagnostic methods for depression is often determined through clinical interviews with doctors using scales such as ICD, DSM, and SDS [31].Previous studies have demonstrated that parental factors, educational background, living standards, as well as exercise and social interaction, can be utilized as auxiliary diagnostic indicators for psychological issue [29,32,33]. In this study, we aim to further explore AI methods for predicting the risk of depression in adolescents, using factors such as exercise, family influence, and lifestyle habits to assist in predicting the risk of depression in adolescents. At the same time, to address the easily overlooked psychological issues of adolescents, design an architecture method that is accessible to ordinary users. In addition, health census data is a valuable database for big data applications, and we will fully utilize health census data as the basic data support in this study.

We have developed an Adaptive Adolescent Depression Assistant (AADA) Predictor that supports online learning, with a core algorithm built upon an automated machine learning (AutoML) mechanism. The AADA can be deployed in the cloud to provide API-based services for adolescent depression risk prediction and screening support using census survey–derived daily-life indicators, enabling scalable risk stratification and follow-up prioritization. Importantly, the AADA is intended to support screening and decision-making workflows rather than to replace clinician-administered diagnosis. We further conduct model interpretability analyses to identify and quantify key factors associated with elevated depression risk. The contributions of this article are as follows:

- We collected and organized NSCH adolescent health survey data (2020–2022) to create a machine-learning dataset. For data processing, we calculated data similarity to assess sample importance and used sampling to form balanced sub-datasets, addressing severe sample imbalance and enhancing model performance.
- Based on automatic machine learning methods, we have improved the AutoML toolkit to make it more suitable for depression risk assessment in adolescents based on daily data. This method enables rapid, adaptive training on diverse data structures without manual parameter tuning. Additionally, we proposed a cloud service framework to improve overall availability.
- We generated various datasets from census data to validate our method. Results show that it significantly aids in assessing adolescent depression risk and supporting screening workflows, rather than providing clinical diagnosis. We also analyzed model interpretability by comparing variable correlations and feature importance. Through localized interpretability, we detailed how different feature values affect outcomes. Based on these findings, we provided recommendations for adolescents’ daily lives and physical activities.

## Materials and methods

In previous studies, there has been significant research on disease prediction, but the use of big data for large-scale mental health risk assessment and screening support remains limited. On one hand, many psychological conditions lack readily available objective biomarkers in routine settings, and obtaining gold-standard labels such as clinician-administered diagnostic interviews at scale is difficult. On the other hand, there are significant challenges in collecting, harmonizing, and processing real-world behavioral and survey data.

To address these challenges, we propose a method that improves data accessibility through simple, survey-based data collection. Additionally, the data preprocessing techniques employed in our approach are designed to improve efficiency and robustness for imbalanced, high-dimensional census-derived data.

### Data resource

Through research, we located relevant study data, which come from the publicly available NSCH datasets in the United States [34]. The NSCH provides rich data on various aspects of children’s lives, including physical and mental health, access to quality medical services, and family, community, school, and social environments. This study used the NSCH datasets from 2020 to 2022, which were obtained from the MCHB website in 2025. For more information about NSCH methods, survey content, and data availability, please refer to the MCHB website.

In the census data from 2020 to 2022, the entries for each set of data amount to 42,777, 50,892, and 54,103 respectively. According to the Topical Variable List, the item ’hhid’ refers to the Unique Household ID, and all of the ’hhid’ values in the three years of the survey are different. The data regarding the three years of survey data and the incidence of depression in children are as shown in Table 1.

**Table 1:** 2020-2022 Census Database Statistics.

Title	2020 Topical Data	2021 Topical Data	2022 Topical Data
Items	42,777	50,892	54,103
Variables	443	463	490
Has Been Diagnosed Depression	2,316	2,605	3,103
	5.41%	5.12%	5.74%
Currently Depression	1,851	2,095	2,498
	4.33%	4.12%	4.62%

From the statistics, we can see that among adolescents aged 0–17, approximately 5%−6% have suffered from depression, and the rate of those currently suffering from depression is about 4.5%. The statistical tables also reveal that the number of data variables in the census data from 2020 to 2022 all exceed 400. After aligning the data, we selected all items with a correlation greater than 0.05 to depression through Pearson correlation analysis.

We selected 30 variables for research from five dimensions: Personal Information, Physical Health; Mental Health; Living/Study; Family, by consulting experts. The specific details are shown in the table. We also calculated the Number of Default Values for each sample. In the presented data, all meet statistical norms, and the quantity of default values in all data does not exceed 4.3% for any item. All selected samples are statistically valid.

See Table in S1 Table. for details about selected variables and data default situation.

### Methods

Despite progress in disease prediction, big data applications in psychological diagnosis and assessment are limited. Previous methods struggle with identifying subtle psychological risks, and managing large-scale survey data remains challenging.. We propose a big data-enabled approach for adolescent depression risk assessment, enhancing data handling and study efficiency.

#### Cloud-based frameworks.

**Fig 1:**
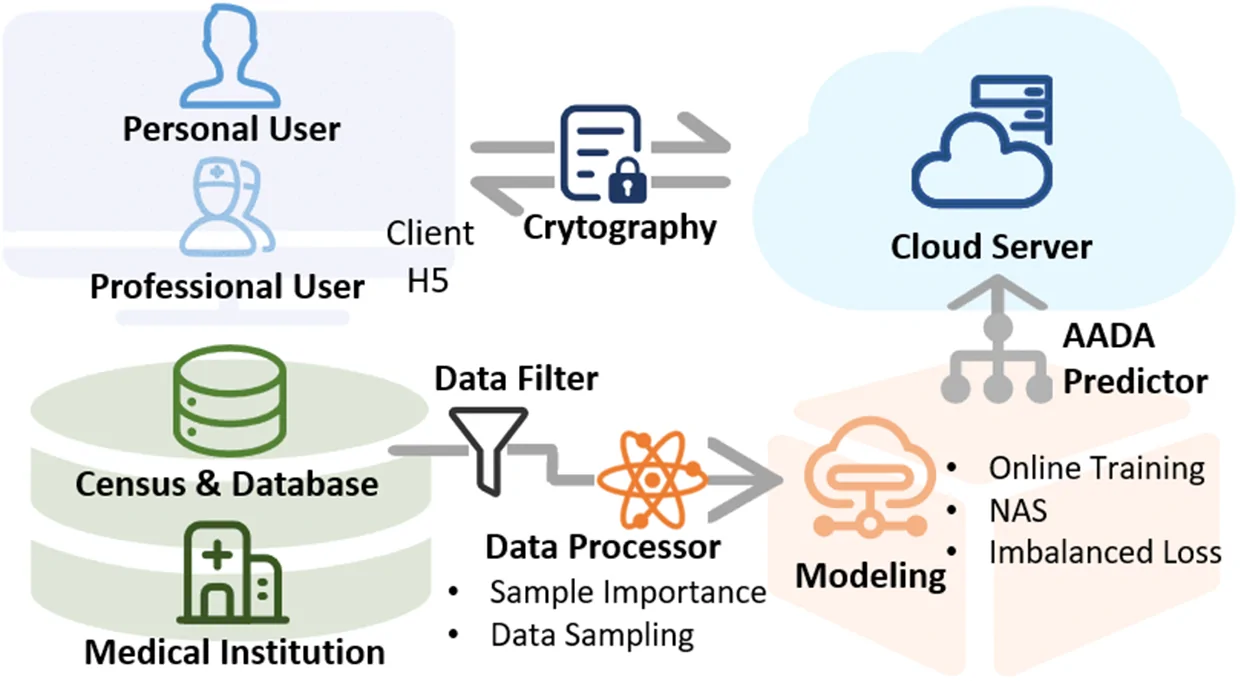
System architecture. Census/survey and authorized institutional data are continuously ingested, filtered, and processed, then used to update the AADA predictor via AutoML with imbalance-aware training. The most recent model is hosted on a cloud server and exposed through encrypted client access for depression risk assessment.

To support scalable deployment and future model updates, we design a cloud-based architecture for the proposed AADA predictor. The framework integrates data preprocessing, AutoML-based model training, and encrypted client-cloud communication. The online update mechanism enables model adaptation when new data become available, while a basic homomorphic encryption scheme is adopted to enhance data privacy. The encryption component is not the focus of this study; therefore, only its basic integration is considered, as shown in Fig 1.

#### Data process.

Targeted Data Augmentation Method for Medical Applications: Data augmentation [35] is a commonly used technique in machine learning for increasing the quantity and diversity of limited data to increase the robustness. Data augmentation methods currently include paraphrasing-based, noise-based, sample-based, and other approaches [36]. Usually, the natural collection volume of positive samples in medical data is significantly lower than that of negative samples. When the amount of data collected in medical samples is small, it is necessary to use data augmentation methods to expand the dataset while ensuring the accuracy of the data. We refer to a hybrid sampling algorithm RFMSE [37] for medical imbalanced data, which combines the Misclassification oriented Synthetic minimum over sampling technique (M-SMOTE) [38] and Edited Nearset Neighbor (ENN) [39]. We propose a targeted data processing process for medical data:

- Adaptive SMOTE Method: In an imbalanced dataset where the negative samples are significantly fewer than the positive samples, we only need to apply SMOTE to the positive samples. Using our proposed implementation approach, the generated data from SMOTE will be labeled as synthetic data.
- Adaptive ENN Method: Based on the data entry scoring and labeling mechanism we proposed, there is no need to remove the under-sampled data generated by ENN. Instead, we simply decrease its importance coefficient in the database.

The original samples featured by quantity and imbalance. The imbalance rate ℝ of samples can be defined as:


$$\begin{array}{c} \mathbb{R}=\frac{\sum_{i=1}^{c}\left|S_{i}-S/c\right|}{S}\times 100\% \end{array}$$ (1)

SMOTE is based on the k-nearest neighbor algorithm. Sample $S_{j\_\text{min }}$ is chosen from each minority class sample $S_{i\_\text{min }}$ to generate a new minority class sample:


$$\begin{array}{c} S_{\text{new }}=S_{i_{-}\text{min }}+\mathrm{rand}(0,1)\left(S_{j_{-}\min}+S_{i_{-}\min}\right),i=1,2,\cdots ,n,j=1,2,\cdots \end{array}$$ (2)

Algorithm uses the misclassification rate of samples as the oversampling rate, rather than the imbalance rate. During the iterative sampling process, the oversampling sampling rate is dynamically updated by using the error classification rate of Random Forest. $M_{mis\_class}$ represents the number of miss classifications *S* for a certain type of sample:


$$\begin{array}{c} M_{mis\_class}=\sum_{i=1}^{n}\left(\mathrm{Class}\left(S_{i}\right)\text{≠}C\left(S_{i}\right)\right) \end{array}$$ (3)

In summary, we suggest using a combination of SMOTE and ENN, where SMOTE is applied only to the positive samples, and ENN-generated under-sampled data is labeled and adjusted in the database based on the entry scoring mechanism. Our parameter dimensions are about 30, and based on previous research [40], the SMOTE method is effective under such dimensions.

**Database with information evaluation scores and relevant labels**. In typical data processing methods, certain samples are often removed to achieve balanced data, which may do harm to the richness of the original data. Since general data augmentation and filtering techniques may not adequately address severe data imbalance issues, we proposed a method CensusFilter to use data with sample scores and a data extraction algorithm. In this algorithm, each data entry is assigned a score which represents the importance. The scores range from 0 to 1, with higher values indicating higher importance. The following formula describes the equation for scoring importance, where $n_{p}$ and $n_{n}$ represent positive samples (which are generally fewer in quantity in this study) and negative samples, respectively. The coefficient λ is a manually defined hyperparameter, and we use default values. The influence of hyperparameters on data sampling will be discussed in subsequent experiments.


$$\begin{array}{c} \theta_{1}=\frac{\lambda_{1}}{\ln(e-1+n_{k})} \end{array}$$ (4)

$$\begin{array}{c} \theta_{2}=\frac{n_{p}}{n_{n}}\cdot \frac{\lambda_{2}}{\ln(e-1+n_{k})} \end{array}$$ (5)

where $n_{k}$ represents the number of data entries in a similar class. In this study, due to the high dimensionality and large volume of the data, using conventional clustering algorithms may result in excessively large clusters. Therefore, DBSCAN method [41] were chosen to implement the clustering functionality for different samples. To handle high-dimensional features of the data source, we select the HDBSCAN [42] implementation. HDBSCAN (Hierarchical Density-Based Spatial Clustering of Applications with Noise) is a density-based clustering algorithm. The “Hierarchical” in its name reflects a key characteristic of this algorithm, which reflects the density between data points and the hierarchical structure of these densities, allowing for the discovery of clusters with different densities. It is also necessary to consider the diversity of data categories; if the data clusters are too large, the sampling coefficient will be reduced. Therefore, we improve the HDBSCAN algorithm by adding a constraint on the maximum number of clusters. We set the parameters $min_{samples}$ = 3 and $max_{samples}$ = 20 to ensure the diversity of sample categories and to prevent excessively low sampling probabilities for samples in the same category. Through calculations, when $\lambda_{1}$=1.35 is used, the minimum importance was limited to $\theta_{1}$=0.4. Meanwhile, in our work, we differentiate between original data and generated data by assigning labels which is 0 or 1. It indicate whether the generated sample is from the original data. Then we have the final formula for the label score.


$$\begin{array}{c} \theta_{gen}=\left\{ \begin{array}{cc} \lambda_{gen}, & n_{gen}\leq n_{p}\\ \frac{\lambda_{gen}n_{gen}}{n_{p}}, & n_{gen}>n_{p} \end{array}\right. \end{array}$$ (6)

#### Prediction algorithm for assessment.

**Traditional machine learning method**: Where $\lambda_{gen}$ is evaluated manually, from 0.1 to 0.2. $N_{p}$ is the number of positive samples. Before the appearance of deep learning, traditional machine learning methods played a leading role in the field of artificial intelligence. These methods utilized statistical techniques to enable computer programs to learn patterns from data and make predictions on new data. Logistic regression and XGBoost are commonly used machine learning methods for classification problems. In our comparative experiments, we will select the simplest logistic regression method as the baseline model.

**Deep learning prediction methods**: One prominent architecture in deep learning is the multilayer perceptron (MLP), which has made deep learning feasible. In our research, we propose an innovative algorithm and use a simple MLP as a comparative model. The MLP consists of multiple layers, including an input layer, hidden layers, and an output layer. We set the number of hidden layers to 3 in this case. Each layer contains 5–20 neurons, which are interconnected through fully connected connections. Additionally, we apply dropout to enhance the model’s robustness.

**Fig 2:**
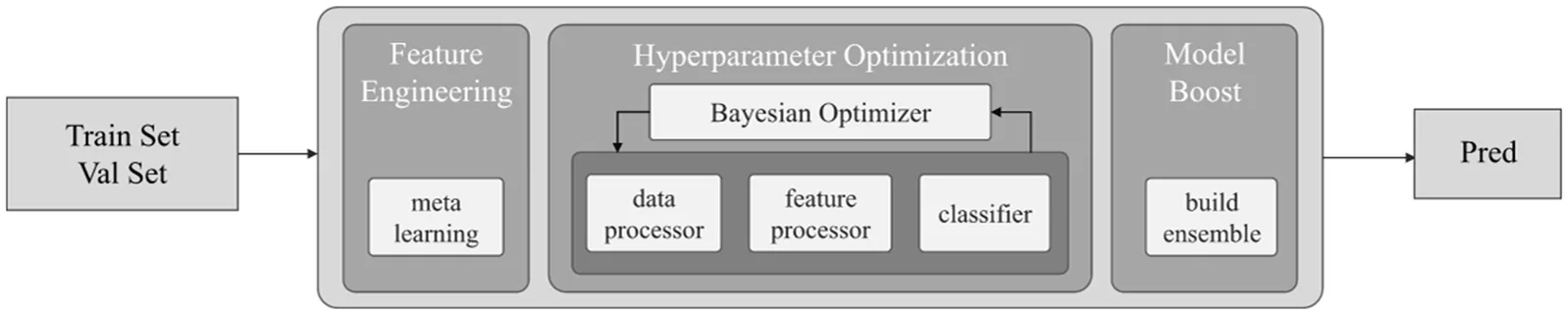
Flowchart of automated machine learning (AutoML) modeling process based on Bayesian Optimization [49] method. The AutoML method includes three core elements: feature engineering, hyperparameter search, and model boost. Feature engineering uses meta-learning [50] methods to induce sample features and recommend models architecture using prior knowledge. Bayesian optimization is a popular method in hyperparameter search. It evaluates the dynamically changing hyperparameters on model performance and improves hyperparameters.

**Sampling combination prediction method based on AutoML**: In previous risk prediction research, machine learning methods such as regression, decision trees, XGBoost, and SVM were commonly used for datasets with multiple data distributions. These methods were suitable because the dimensions and data were not complex, making them easy to handle. However, for our datasets, which has a large volume of data and multiple analysis features, it is necessary to customize the prediction models based on the data because manual modeling will be time-consuming and labor-intensive. To solve this, we introduce the concept of AutoML for automated prediction model building. AutoML proposes the idea of adjusting the model based on the feedback of loss obtained during training on different datasets. Model adjustments are performed within a predefined search space. Such search actions are typically divided into two categories: model structure search [43] and hyperparameter search [44,45]. We choose the mainstream method in AutoML methods, Bayesian optimization [46]. As an example, the Auto-Weka series [47] and Auto-Sklearn series [48] employ Bayesian optimization to select combinations and parameters, achieving better performance on datasets. However, considering the limitations of AutoML in handling large-scale data, we draw inspiration from Boosting and Rainbow methods and perform multiple sampling on the scored label data to create different datasets. These datasets are then used to build multiple models, which are combined into an Advanced Predictor for prediction purposes. The whole process is illustrated in Fig 2.

In order to improve management, we have also enhanced the loss calculation method, taking inspiration from the Focal Loss calculation approach for imbalanced data. Considering binary classification and cross-entropy loss:


$$\begin{array}{c} L_{ce}=-\sum_{i=1}^{n}y_{i}log(p_{i}) \end{array}$$ (7)

Building upon cross-entropy loss, Focal Loss [51] introduces a weighting factor α to reduce the impact of positive samples on the loss when the predicted probability value *p* for positive samples is greater than 0.5. The Focal Loss can be formulated as follow:


$$\begin{array}{c} \begin{array}{c} \alpha_{t}=1-p_{t}\\ FL(p_{t})=-\alpha_{t}^{\gamma }\log(p_{t}) \end{array} \end{array}$$ (8)

Where $\alpha_{t}$ is the coefficient of the basic cross entropy loss function, which balances the impact of a small number of samples and a large number of samples on model performance, while gamma is the hyperparameter that controls the magnitude of the coefficient’s impact on the entire loss function. However, the Focal Loss is original designed for object detection task in multi-class discipline. The final loss calculation method we designed shows in (9). In our implementation of automated machine learning, we also incorporate data volume weighting as follows, where *N* represents the total sample size in the current data extraction, the $N_{b}$ is the training batch size, and $n_{t}$ represents the data volume for that class. This design significantly improves the balance of the calculations with respect to the samples. λ ranges from 0 to 3.


$$\begin{array}{c} L=-{\frac{1}{N}}_{b}\sum_{i=1}[(\frac{n_{t}}{N}{)}^{\lambda }\cdot y_{i}\cdot log(p(y_{i}))+(\frac{N-n_{t}}{N}{)}^{\lambda }\cdot (1-y_{i})\cdot log(1-p(y_{i}))] \end{array}$$ (9)

**Fig 3:**
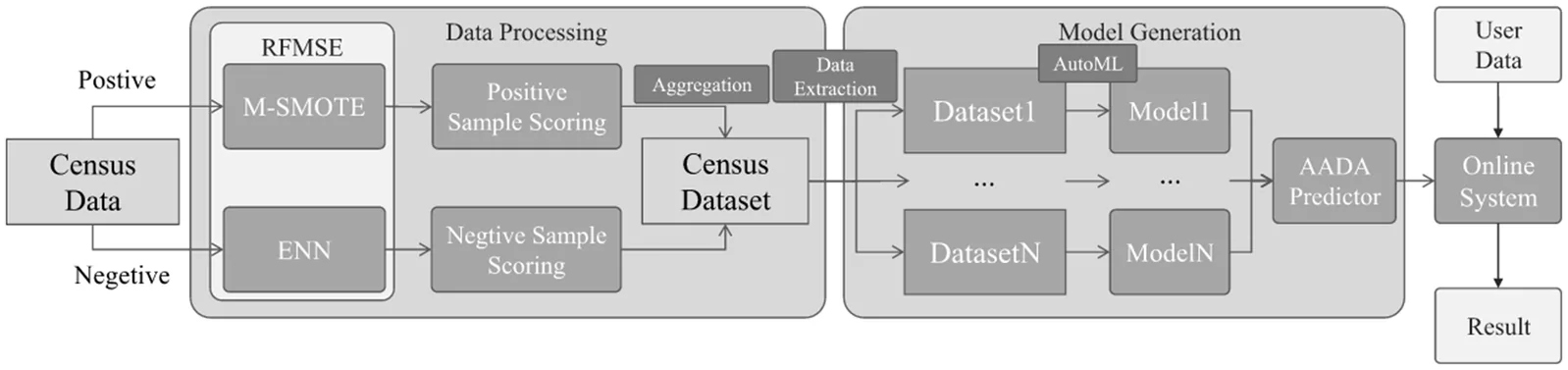
The workflow diagram of the system. The workflow of the entire system contains of data process and modeling. The original data is divided into positive and negative sample, while different meta methods are employed for different categories. After aggregation, different batch subsets are sampled by extraction. Adaptive modeling method for train subsets generates diverse single model predictor, which proposes for the boosted predictor (AADA Predictor).

**Overall pipeline of our method**: The working pipeline of the whole system is illustrated in Fig 3. Our overall methodology is to form a machine learning usable database with data information through customized data processing by CensusFilter. The model generator adaptively establishes classification models for different training data, ultimately forming an ensemble model. The final model (the AADA) has strong robustness against samples from imbalanced data ratios.

### Interpretability of prediction methods

Shapley value [52], a widely used method in cooperative game theory, offers stronger interpretability for machine learning models than general correlation analysis methods. It describes the contribution of each feature’s importance in diverse contexts. Initially, SHAP packages [53], which are grounded in the Shapley value framework, were primarily designed to elucidate the feature importance for tree-based models, as well as linear and nonlinear models. However, with the advancement of research, these packages have now been extended to accommodate the interpretation of feature importance in deep neural network computations. In this study, we employ the SHAP package for feature analysis.

It should be noted that the initial technical roadmap of SHAP was predominantly focused on supporting linear and tree-based classification methods. Consequently, most prior research utilizing SHAP tools [54–56] has been limited to basic machine learning models for analysis. In our work, we select the XGBoost method, which demonstrated the most superior performance in our preliminary basic experiments, and the AADA method, which achieved the best performance in our specific experimental context, for comparative analysis.

### Experimental setup

We will conduct four sets of experiments to validate the effectiveness of the proposed method and, to some extent, analyze the causal factors contributing to adolescent depression based on the content of the existing database.

**Basic data preparation and missing-data processing**: We first performed statistical correlation analyses on the basic data to demonstrate that the data source is statistically reliable and interpretable. Since the census database contains missing values and some machine learning methods cannot handle missing data directly, we imputed missing values using different methods and selected an appropriate imputation strategy for subsequent experiments based on empirical results. In addition, we independently constructed a balanced test set by sampling unbiased data from the original dataset, with a total size of 5,000 instances, including 2,500 positive samples and 2,500 negative samples. Besides, we choose 1,000 positive samples from balanced dataset and another 10,000 negative samples from original data to construct an unbalanced test dataset. Both test sets were reserved for evaluating the baseline and proposed methods, with an overall training-to-test ratio of 8:2. All class-imbalance processing was restricted to the training data: M-SMOTE was used to generate additional positive samples, while ENN and CensusFilter-based scoring and sampling were applied to the negative samples.

**Baseline methods**: In our experiment, each method was carried out three times, and different random seeds were used to get the average results. Among them, **Decision Tree**, **XGBoost** and **Logistic Regression** do not need to set additional hyperparameters, while in **SVM**, linear kernel was used and set the penalty coefficient to 1. **DNN** parameter setting: 3 hidden layers with 20 neurons in each layer, and the dropout rate of the last layer is set to 0.3, learning rate *lr* = 0.001.

**Our method**: We first preprocess the data and compute scores using the CensusFilter method, and then perform multiple rounds of sub-dataset sampling. The AutoML component was implemented using AutoKeras 1.0.20, with Bayesian optimization employed for neural architecture and hyperparameter search. Following the training procedure shown in Fig 2, the sampled models are finally integrated via a boosting strategy to form the AADA predictor. We adopt five different strategies to automate training and obtain models; these strategies are evaluated individually, and we finally evaluate the overall AADA predictor.

**Risk prediction reliability and interpretability**: The sigmoid probability outputs before thresholding were retained to evaluate calibration performance using reliability curves, Brier score, and expected calibration error (ECE). SHAP analyses were further conducted using contribution values, summary plots, and scatter plots to interpret feature contributions. We also performed SHAP analyses stratified by major feature categories to examine how different discrete feature values influence model predictions.

**Computational environment**: All experiments were conducted on an Intel NUC 11 system equipped with an Intel Core i7-11700B CPU, 32 GB of system memory, and an NVIDIA GeForce RTX 3060 GPU. The software environment consisted of Python 3.9, AutoKeras 1.0, TensorFlow 2.15, and Keras 2.15. All experiments were repeated three times with the random seed fixed at 42.

## Results

### Data and analysis

**Fig 4:**
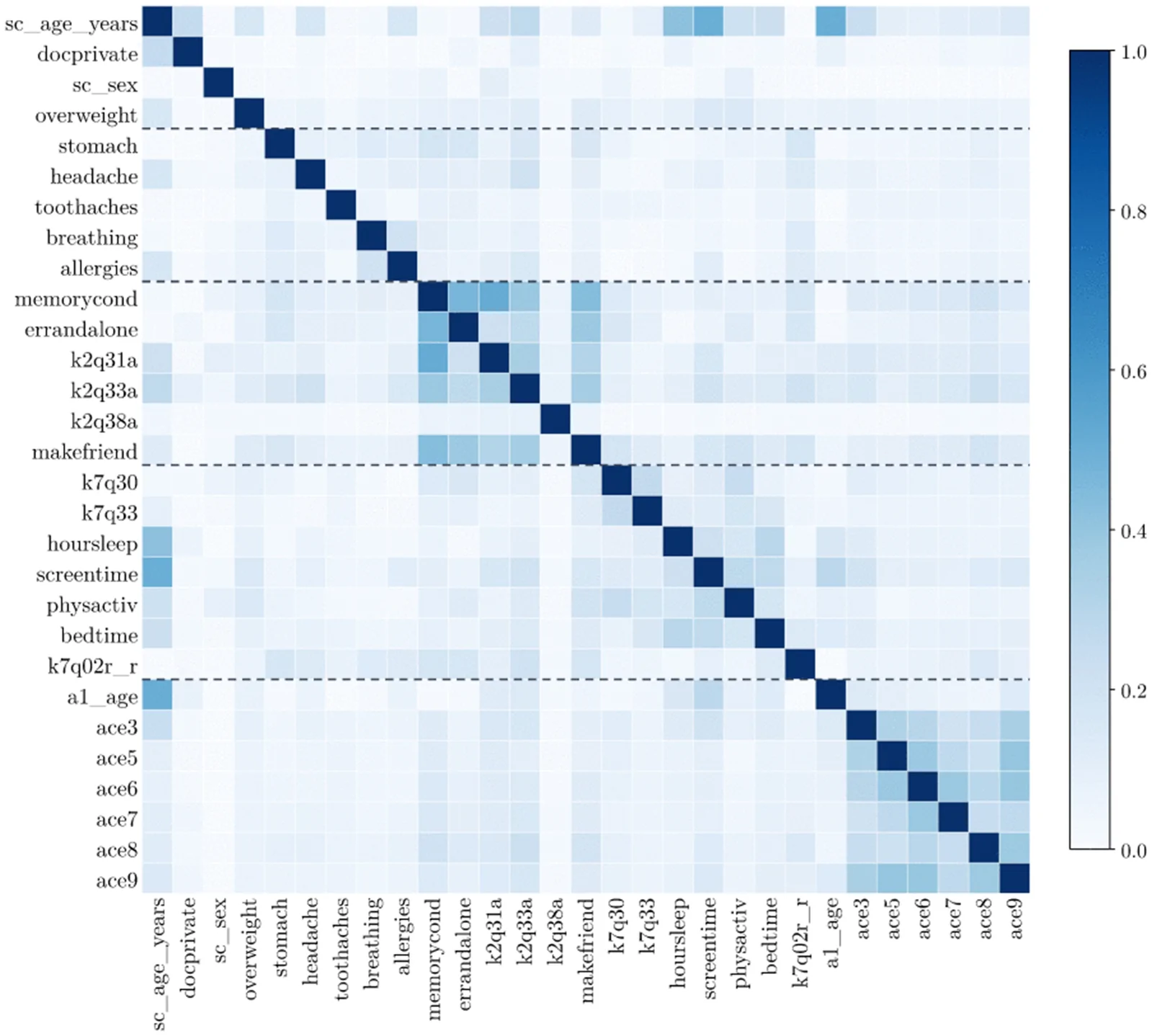
Heatmap of correlation between different dimensions. The figure is divided into different major categories by a black dashed line. Psychological issues are highly correlated with intra-group elements of the living environment. The correlation distribution of some features related to depression (e.g., k2q32) is relatively uniform, except for Mental Health. The lack of significant correlation in other items underscores the complexity of the original data and the need for effective data processing methods to decipher it. (With all the factor *p* < 0.01).

**Fig 5:**
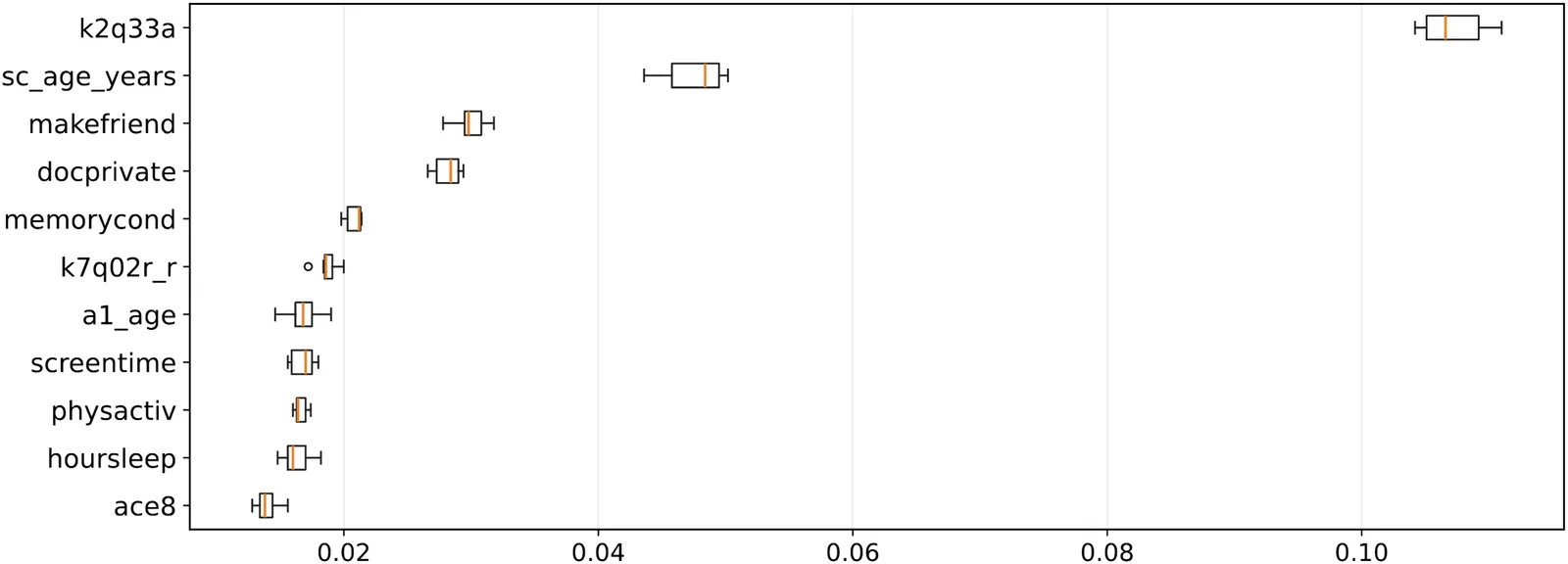
Permutation importances of the top 11 variables. The feature-importance dot plot shows the 11 most important variables for adolescent depression risk assessment. Variable importance is measured by two classifiers: RandomForest and XGBoost. The analysis indicates that, compared to other factors, mental health and daily activities contribute more to the model predictions, consistent with prior studies.

Basic data analysis is conducted on the original data, such as correlation analysis and feature importance analysis, to demonstrate the usability of the data. We show the result of the correlation analysis in Fig 4, and feature importance in Fig 5.

A large amount of missing data may affect the performance of machine learning methods [57], although there are methods that support situations where there are missing values in the data. In order to achieve better results, some methods for handling missing data samples have been proposed, including deleting samples with missing data, replicating such samples to supplement insufficient quantities, and supplementing missing values [58]. Among them, there are many studies on missing value filling methods, such as using fixed values such as mode, mean, and special values, or using statistical methods such as linear interpolation, KNN interpolation, and random forest interpolation for missing value filling [59]. In our basic experiment, we selected some filling methods for comparison, the results of different methods for missing value imputation shows in Table 2. We chose the KNN method to fill in all missing values in the original training and testing sets, and in subsequent experiments, we also used the filled dataset. However, it is worth noting that mode filling is very efficient and performs only slightly lower than KNN one. It is recommended to use this method when dealing with larger scale data.

**Table 2:** Performance of different missing value filling methods. ACC values are reported with an uncertainty of ±0.05 across three runs.

Model	Constant(−1)		Mode		Mean(integer)		KNN		RandomForest	
	ACC(%)	AUC	ACC(%)	AUC	ACC(%)	AUC	ACC(%)	AUC	ACC(%)	AUC
Decision Tree	70.90	0.71	71.72	0.71	70.45	0.70	71.29	0.71	70.27	0.70
XGBoost	73.06	0.73	72.93	0.73	72.81	0.71	73.06	0.73	72.66	0.72
Logistic Regression	70.05	0.69	72.74	0.69	72.58	0.68	72.91	0.68	72.18	0.68

### Baseline methods

We conducted preliminary testing for various basic models on the original dataset, and the results are shown in Table 3. In our experiment, each method was carried out three times, and different random seeds were used to get the average results. Among them, **Decision Tree**, **XGBoost** and **Logistic Regression** do not need to set additional hyperparameters, while in **SVM**, linear kernel was used and set the penalty coefficient to 1. **DNN** parameter setting: 3 hidden layers with 20 neurons in each layer, and the dropout rate of the last layer is set to 0.3, learning rate *lr* = 0.001. After experiments, we found that when the epoch is set to about 10, loss value tended to be stable.

**Table 3:** Preliminary testing of the predictive ability of the basic modesl on the original dataset. Including we deploy Decision Tree, XGBoost, logistic regression, SVM, and a simple multi-layer neural network. At the same time, we also extracted the test set as a sample balanced version, and the performance of XGBoost and neural network models was acceptable.

Model	Imbalanced Testset		Balanced Testset	
	ACC(%)	AUC	ACC(%)	AUC
Decision Tree	93.62±0.05	0.73	71.29±0.05	0.71
XGBoost	95.71±0.05	**0.73**	**73.06**±0.05	**0.73**
Logistic Regression	95.56±0.05	0.69	72.91±0.05	0.68
SVM	94.80±0.05	0.53	52.62±0.05	0.53
DNN	**95.87**±0.05	0.70	72.79±0.05	0.72

### Our methods

**Fig 6:**
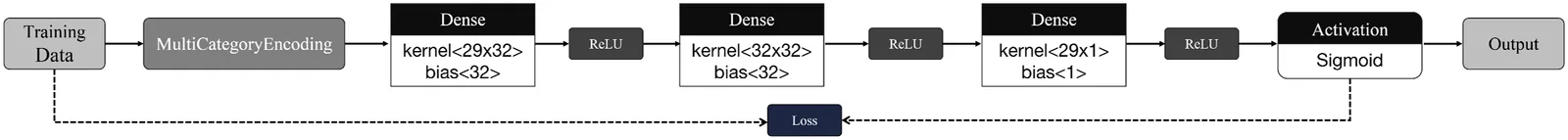
Result of the optimized DNN architecture.

We conducted experiments using our proposed method, which involved scoring the data via a scoring system and building models with our adaptive approach. In the final experiment, we performed five rounds of sampling, each time saving the subsampled dataset as a new training dataset. Our test set is the same as the dataset from the previous underlying experiments. The results are shown in Table 4. In terms of results, although the accuracy of judgment decreased in the unbalanced dataset, the AUC results were significantly improved, which indicates that the processing power of the model in the unbalanced dataset has been improved. Because in the unbalanced data set, the proportion of negative samples is as high as 95%, and if all the outputs are negative, the accuracy will be as high as 95%, but this obviously does not mean that the model is a very poorly effective model. The test results on the data equilibrium test set show that the accuracy of all the tests is significantly improved compared with the basic model test, and the AUC of the experiment also improves to more than 0.92. The experimental results show that our work is meaningful. The models automatically generated under different training sets may vary slightly, but the final basic structure of the backbone network is shown in Fig 6. We also investigated the impact of custom loss on model performance.

**Table 4:** Experiment for different loss strategy in our proposed method. Focal Loss* is the improved version for data imbalance based on focal loss. The Boosted is the final aggregation model.

Model	Train Set Samples	Loss Strategy	Imbalanced Testset		Balanced Testset	
			ACC(%)	AUC	ACC(%)	AUC
1	19,687	CrossEntropy	89.63±0.05	0.92	82.67±0.05	0.93
2	20,673	CrossEntropy	90.35±0.05	0.93	84.37±0.05	0.93
3	20,132	CrossEntropy	91.12±0.05	0.94	84.53±0.05	0.94
4	20,132	Focal Loss	88.97±0.05	0.91	83.57±0.05	0.93
5	20,132	GHM Loss	89.93±0.05	0.92	85.72±0.05	0.93
6	20,132	Focal Loss*	**93.12**±0.05	**0.95**	**86.51**±0.05	**0.95**
Boosted	–	–	90.50±0.05	0.94	86.38±0.05	**0.95**

In the final, we conducted comparative experiments between the baseline predictors and our proposed adaptive modeling approach, utilizing a synthetic training dataset processed with the CensusFilter data handling method. The training dataset was synthesized from two instances of CensusFilter sampling, totaling 40,289 data entries. The test set was chosen to be the same balanced dataset used previously. The results are shown in Table 5. Initially, the implementation of our proposed **CensusFilter** data processing method resulted in a marked enhancement in the performance of both fundamental machine learning algorithms and deep neural network approaches, thereby validating the efficacy of our data processing technique. Subsequently, our **AADA** Predictor was found to surpass all other methods when evaluated on the same dataset. It should be noted that certain baseline methods outperformed the experimental data presented in Table 5. This divergence can be ascribed to the fact that our prior experiments relied on a dataset derived from a singular sampling approach, in contrast to the current experiment, which capitalized on a training set amalgamated from two rounds of sampling.

**Table 5:** Results of method comparison under the data from CensusFilter generation on balanced testset.

Model	Precision(%)	Sensitive(%)	Accuracy(%)	F1(%)
XGBoost	87.03±0.05	77.24±0.05	85.91±0.05	85.82±0.05
SVM	87.12±0.05	79.77±0.05	86.42±0.05	86.33±0.05
DNN	87.01±0.05	80.72±0.05	86.96±0.05	87.12±0.05
Single Predictor	87.95±0.05	81.95±0.05	87.28±0.05	87.18±0.05
Boosted Predictor (AADA)	**88.25**±0.05	**82.50**±0.05	**87.92**±0.05	**87.80**±0.05

### Calibration of risk predictions

Calibration was evaluated on the original imbalanced test set (*n* = 29,447), where the observed depression prevalence was 5.34%. Before correction, the sigmoid probabilities showed limited calibration, with a Brier score of 0.0557 and an ECE of 0.0847. After applying training-prior probability correction, both metrics were substantially reduced (Brier score: 0.0327; ECE: 0.0181), indicating improved agreement between predicted probabilities and observed outcomes. These results suggest that correcting the sampling-induced prior shift effectively improved the reliability of risk predictions.

### Interpretability of risk predictions

**Fig 7:**
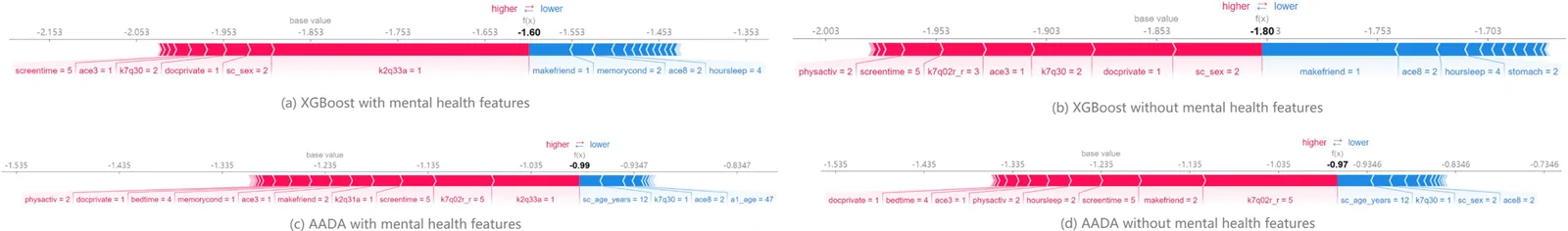
Game of contribution values of feature variables. Taking subplot (a) as an example, it describes 10 characteristic games with the greatest impact on model prediction, led by k2q33a (Anxiety). The red bar represents a positive contribution, while the blue bar represents the opposite.

Fig 7 shows the contribution of different features to the predicted risk outcomes. When the value of **k2q33a** is 1 (previously or currently suffering from anxiety), it makes the greatest contribution to the model prediction. This is also consistent with the previous psychological findings that depression often accompanies anxiety.

**Fig 8:**
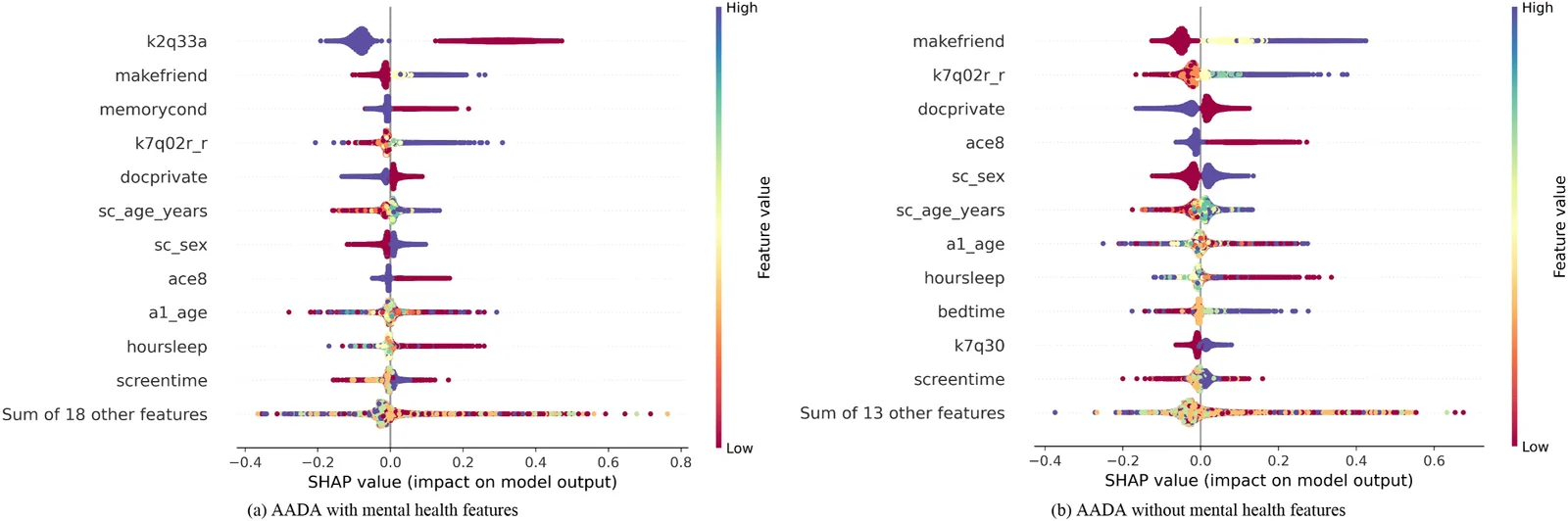
SHAP value summary of AADA method. Subplot (a) is the method under all features we select. And (b) is excluding categories of psychological issues.

Fig 8 visualizes the contribution of each feature when using the AADA model for prediction. We can see that when the **k2q33a** feature is low (value 1), the contribution is greater than when the feature is high (value 2, no history of anxiety). This is in line with previous psychological findings: it can be explained that adolescents who have previously suffered from anxiety tend to have a greater tendency towards depression. The low contribution of having no history of anxiety means that it cannot be interpreted as a decrease in the probability of developing depression. In longitudinal analysis, we can notice that for example, **k7q02r_r** and **docprivate** do not rank highly in the correlation analysis in Fig 4, and their ranking in the importance analysis in Fig 5 is different. However, the Shapley values demonstrate a dominant role, which means that specific values of certain features will make important contributions to model predictions.

We also selected different features for analysis based on the major categories. In the category of psychological problems, in addition to the **k2q33a** mentioned earlier, the average contribution of game values for **memorycond** with a value of 1 (memory difficulty) and **k2q31a** with a value of 1 (history of ADD/ADHD) is significantly higher than the negative contribution when the value is 2. For the **Living/Activity** category, a **k7q33** value of 5 (almost no participation in activities and exercise) and hourly sleep values of 1, 6, and 7 (less than 6 or more than 10 hours of sleep per day) will have a significant positive impact on judgment. A **physactiv** value of 4 (engaging in at least 60 minutes of physical activity every day) will contribute negatively to the judgment. The different values in each of the above features can better explain the basic rules followed by the model’s judgments in the game.

Overall, the SHAP analyses suggest that the proposed models rely on clinically plausible daily-life indicators and comorbid mental-health variables when available. These findings support our approach for scalable risk stratification and screening-oriented decision support using census/survey data, rather than replacing clinician-administered diagnosis. **Note:** Results are based on NSCH and may not generalize to other regions; the pipeline can be re-trained on local datasets.

## Discussion

Adolescent depression is frequently overlooked by guardians due to youths’ limited ability to recognize and articulate symptoms, compounded by the scarcity of pediatric programs that provide relevant training [60]. Although diagnostic criteria broadly parallel those for adults, developmental stage can substantially shape symptom presentation, and many children and adolescents struggle to understand and communicate their emotional states [61]. While machine-learning approaches using EEG [62], MRI [63], or facial-expression capture [64] can achieve high accuracy, these methods typically presuppose suspected psychological problems prior to data acquisition—an assumption that often fails in real-world family contexts. Recent work predicting youth mental health from daily-life and behavioral data reports accuracies of 0.7–0.9 and AUCs of 0.75–0.95 [29,65,66], yet many studies emphasize data collection/processing, provide limited methodological insight, and are described too vaguely for reliable reproduction; overall, existing evidence suggests such models are not yet ready for clinical prognosis [67]. Accordingly, this study aims to develop a depression risk model based on objective, everyday factors to support early risk identification and prevention of adolescent depression within family settings

### Analysis and findings

In the preparation stage, we found that the depression incidence rate was higher in adolescent girls than boys, which is consistent with prior findings that pubertal hormonal changes may increase depression risk in females [68]. Correlation analyses also showed that depression was associated with other conditions, particularly anxiety with a Pearson correlation of *r* = 0.552, consistent with evidence that depressive and anxious symptoms often co-occur and relate to broader health problems [69]. Prior studies also suggest associations between depression risk and factors such as ASD-related characteristics and somatic symptoms [70,71], which motivated their inclusion as candidate predictors. To align with practical screening needs, we evaluated whether the system could predict depression risk using only objective daily-life factors. After removing mental-health comorbidity variables from the inputs, the model still achieved an accuracy of 0.78±0.02 and an AUC of 0.88±0.01, indicating that daily-life indicators contain useful predictive information; however, these results reflect technical validity and do not establish clinical effectiveness or improved outcomes in real-world care.

Prior studies have reported clear associations between adolescent depression and modifiable daily-life factors, particularly physical activity. Regular physical exercise may improve psychological well-being and alleviate anxiety and stress [72]. Evidence also suggests that exercise is associated with improved mental health outcomes [73], and community data indicate an inverse association between physical activity and depressive symptoms [74]. Consistent with these findings, our analyses of Living/Sport variables show that lifestyle habits and physical activity are associated with depression risk and contribute to AADA predictions.

The number of input parameters may limit usability in practice. Questionnaire length and design can influence response quality [75], and mental health assessments can be time-intensive [76]. These considerations suggest that the model would benefit from further refinement to reduce the number of variables. Future work will investigate variable selection strategies to identify a smaller set of inputs while maintaining predictive performance.

### Interpretation of model interpretability

Pearson correlation analysis was first conducted to examine the associations between variables and depression outcomes. The XGBoost model achieved the best performance among baseline models and was selected for comparison with the proposed AADA model. We further employed SHAP values to interpret feature contributions in both models. The rankings of important features showed considerable overlap across correlation analysis, XGBoost importance, and SHAP results, while slight differences were observed due to variations in model mechanisms, feature interactions, data distribution, and multicollinearity, where different models may capture distinct aspects of feature contributions.

Feature importance, which is automatically computed during model training, delineates the average contribution of each feature across the entire dataset utilized in training. The adoption of SHAP values for model interpretability analysis unveils the degree to which different features within a group or an individual data sample contribute to the outcome determination. This approach facilitates a more granular level of interpretability, enabling a focus on the specific influence of particular feature values within a sample cluster on the judgment outcome. Thus, our application of local interpretable model-agnostic explanations provides a more intuitive and quantitative assessment of features, thereby enhancing the confidence in our recommendations for the prevention of adolescent depression.

**Fig 9:**
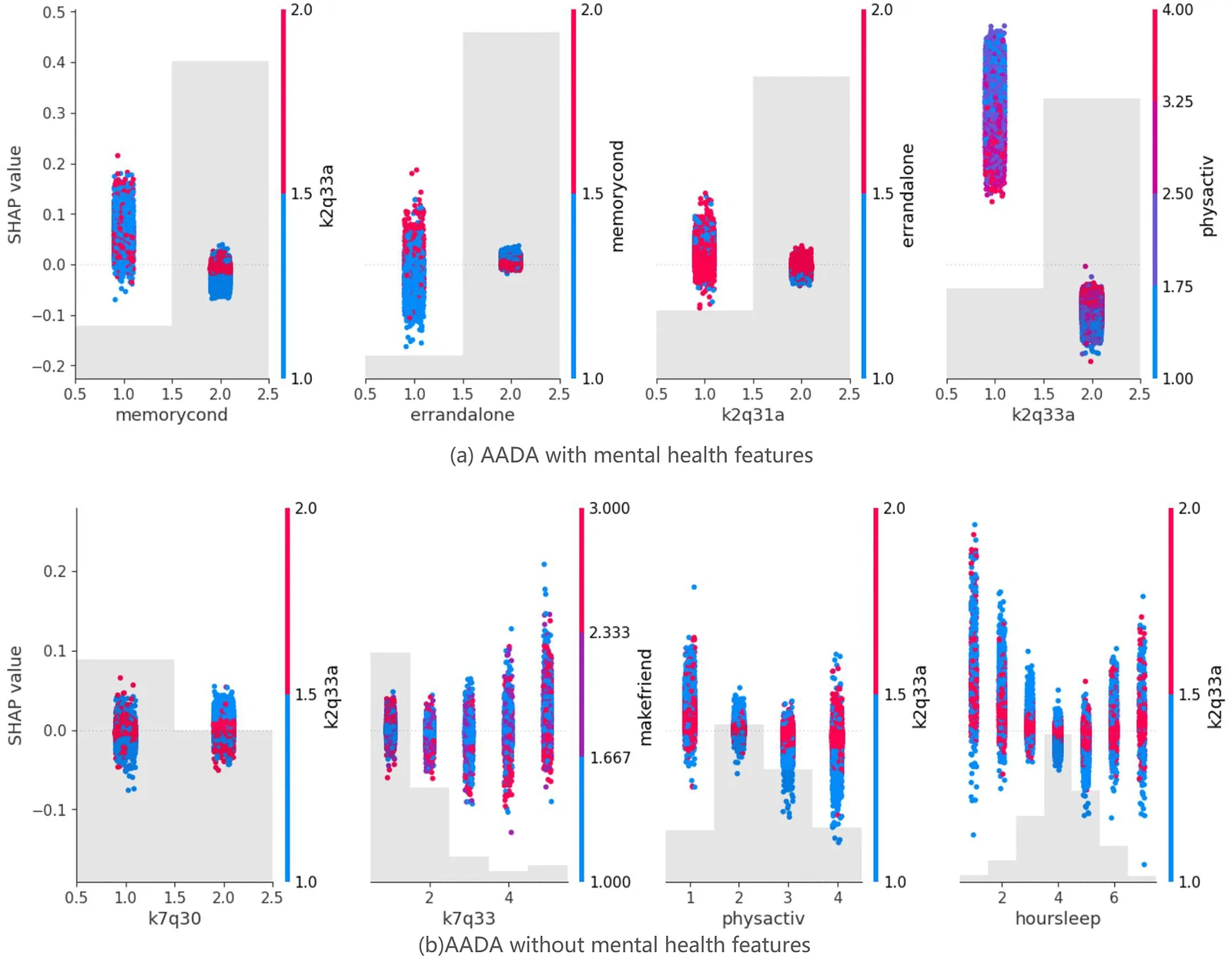
Scatter plot of SHAP value for individual feature. Each point represents one sample; the y-axis shows the SHAP value and the x-axis the feature value, with color indicating an interacting/related feature. For example, in (a) the dependence of errandalone varies with memorycond, suggesting that discrepancies between cognitive difficulty and daily functioning may be informative.

The SHAP-based interpretability analysis suggests that several modifiable daily-life factors are strongly associated with the model’s depression-risk predictions in Fig 9. Very low weekly physical activity is linked to higher predicted risk, while frequent exercise of more than five times per week is associated with lower risk. Sleep duration also shows a clear effect, with both insufficient and excessive sleep increasing predicted risk, underscoring the potential value of regular sleep routines. These findings support promoting a balanced lifestyle that includes regular physical activity and healthy sleep as broadly relevant preventive guidance, but they should be interpreted cautiously because SHAP explanations primarily enhance model transparency and generate data-driven hypotheses and do not by themselves establish clinical actionability or validate intervention effectiveness in real-world practice.

### Ethical considerations and safeguards

This study uses a publicly available dataset that was previously deidentified and approved for academic research, released by the U.S. Census Bureau under approval number CBDRB-FY21-POP001–0161. Given the sensitivity of adolescent mental-health information and the risks of algorithmic labeling, any real-world use should occur under clinical oversight. Outputs should be treated as screening signals rather than diagnoses, with clear referral pathways and safeguards to reduce misclassification, stigma, and unintended harm. In addition, potential biases arising from demographic, cultural, or socioeconomic differences in the underlying data should be carefully considered, and continuous evaluation is needed before broader deployment.

### Limitation

There are still some limitations in this study. All our current data are sourced from the cross-sectional data of the NSCH census spanning 2020–2022. These data do not permit further time-series tracking. Consequently, our study cannot ascertain the causal relationship between variables and outcomes; we can only predict the outcomes after the variables have been processed through the deep neural network. Moreover, we cannot validate whether the variables that need improvement, as suggested by the system, could result in meaningful depression risk reduction in real-world clinical outcomes. As our data are derived from the U.S. census, the findings based on this dataset might only be applicable to local populations, same ethnicity groups, or those with similar cultural and temporal backgrounds. As such, it may not represent adolescent samples from all global and temporal backgrounds. However, a distinctive feature of our designed system is that it obviates the need for laborious feature engineering, enabling rapid model generation and analysis with new data.

Currently, the system for risk prediction and determination provides only two classification results: normal and at-risk of depression. We plan to enhance it with a comprehensive scoring mechanism, including quantitative risk assessment, to offer risk prevention advice to individuals or guardians, highlighting the need to monitor and improve specific variables. Additionally, the census contains some samples with missing entries, which certain model computations cannot accept. Our literature review and basic experiments suggest that both KNN interpolation filling and mode filling can achieve good results, though KNN has better performance but incurs more computational overhead. These conclusions are based on limited data, and we recommend conducting benchmark experiments to determine the best method in practical applications.

As the model is based on census data, it may not accurately capture the psychological state of any given adolescent individual. This inherent limitation underscores the need for clinical experiments to validate its functionality and feasibility in practice. Consequently, the system should currently be regarded strictly as an auxiliary tool; final clinical decisions must not be based directly on its output alone.

### Intended application

This system is intended as a screening aid that integrates physical and psychological indicators to produce a binary estimate of depression risk in adolescents. The output represents a statistical likelihood rather than a definitive diagnosis and is designed to support—rather than replace—clinical judgment by prioritizing individuals for further evaluation. A practical deployment scenario is within secondary-school mental health assessments: brief questionnaires can be used to flag a smaller high-risk subgroup for targeted follow-up alongside routine screening. With adolescent assent and parent/guardian consent, newly collected data may be incorporated for ongoing model updating, helping maintain performance while meeting ethical requirements.

## Conclusion

In our work, we explored the possibility of using census data for identifying adolescent depression risk and proposed a framework for risk prediction through general survey data, including an innovative data processing method and prediction models. Our methodology outperforms traditional machine learning and general deep learning approaches in depression risk assessment. It can support adolescent mental health screening through multidimensional questionnaires and assist early risk identification and prevention efforts. Through data correlation and interpretability analysis, important factors associated with adolescent depression risk can be identified, providing insights for targeted support. The cloud-based training framework enables future model updates and adaptation to new data. Further clinical implementation and outcome evaluation are needed to validate and refine the framework.

## Supporting information

S1 Table Overview of selected variables and data default values situation. (PDF)
