# Supplementary material for: Census-based adolescent depression risk assessment: A novel method and reflections
Source: PLoS One. 2026 Sep 15;21(9):e0357661. doi: 10.1371/journal.pone.0357661 (PMC13577525; doi:10.1371/journal.pone.0357661)
Supplement: Supplementary file 1 [file pone.0357661.s001.pdf]

S1 Table. Overview of selected variables and data default values situation

Variable Category	Variable	Explanation	Number of Default Values 2020	2021	2022
Personal Information	sc_age_year	Age of the Child	-	-	-
	docprivate	Child Spoke with Doctor Privately	186	140	168
	sc_sex	Sex of Selected Child	-	-	-
	overwhight	Identified as Overweight	173	234	275
Physical Health	stomach	Difficulty Stomach Past 12 Months	209	383	432
	headache	Headaches	99	114	166
	toothaches	Difficulty Toothaches Past 12 Months	223	349	407
	breathing	Difficulty Breathing Past 12 Months	80	146	195
	allergies	Allergies	63	107	109
Mental Health	memorycond	Difficulty Concentrating, Remembering	255	195	253
	errandalone	Difficulty Doing Errands	182	148	199
	k2q32a	Depression	114	166	257
	k2q31a	ADD/ADHD	188	283	364
	k2q33a	Anxiety	144	198	278
	k2q38a	Tourette Syndrome	151	230	284
Living/Activity	makefriend	Difficulty Making or Keeping Friends	614	577	485
	k7q30	Sports Team or Sports Lessons -Pasr 12 Months	494	409	506
	k7q33	How Often Attend Events or Activities	450	350	487
	hoursleep	Hours of Sleep	568	464	478
	screentime	How Much Time Spent with TV, Cellphone, PC	770	959	990
	physactiv	Exercise, Play Sport, or Physical Activity 1 Hour	428	338	455
	bedtime	Go to Bed at Same Time	538	770	777
	k7q02r_r	Days Child Missed School - Illness or Injury	415	351	447
Family	a1_age	parental age	1296	1607	1692
	ace3	Parent Divorced	1484	1887	2097
	ace5	Parent Time in Jail	1577	2025	2258
	ace6	Adults Spal, Hit, Kick, Punch Others	1607	2051	2306
	ace7	Victim of Violence	1612	2064	2286
	ace8	Lived with Mentally Ill	1632	2072	2313
	ace9	Lived with Person with Alcohol/Drug Problem	1630	2029	2307
