# Supplementary material for: Census-based adolescent depression risk assessment: A novel method and reflections
Source: PLoS One. 2026 Sep 15;21(9):e0357661. doi: 10.1371/journal.pone.0357661 (PMC13577525; doi:10.1371/journal.pone.0357661)
Supplement: Supplementary file 2 [file pone.0357661.s003.pdf]

Response Letter for Editor

Response to Editor

1.Please ensure that your manuscript meets PLOS ONE's style requirements, including those for file naming.

- We have made the requested corrections to the author information on the title page. Please note that the corresponding author has been changed due to a change in affiliation. No authors have been added or removed; only affiliation details have been updated.

2. Please note that PLOS ONE has specific guidelines on code sharing for submissions in which author-generated code underpins the findings in the manuscript. In these cases, we expect all author-generated code to be made available without restrictions upon publication of the work. Please review our guidelines at:
https://journals.plos.org/plosone/s/materials-and-software-sharing#loc-sharing-code
and ensure that your code is shared in a way that follows best practice and facilitates reproducibility and reuse.

- We will release the source code on GitHub upon acceptance of the paper, and we have now placed the minimized code in the footer of the homepage.

3.We note that the grant information you provided in the 'Funding Information' and 'Financial Disclosure' sections do not match. When you resubmit, please ensure that you provide the correct grant numbers for the awards you received for your study in the 'Funding Information' section.

- The project has been finalized; therefore, we removed the project-related information from the revised manuscript.

4. Thank you for stating the following financial disclosure:
"This research was funded by Research topic on teaching reform in higher education 551 institutions in Jiangxi Province in 2022 grant number: JXJG-22-16-15."
Please state what role the funders took in the study. If the funders had no role, please state: "The funders had no role in study design, data collection and analysis, decision to publish, or preparation of the manuscript."
If this statement is not correct you must amend it as needed.
Please include this amended Role of Funder statement in your cover letter; we will change the online submission form on your behalf.

- The project has been finalized; therefore, we removed the project-related information from the revised manuscript.

5.Please note that funding information should not appear in any section or other areas of your manuscript. We will only publish funding information present in the Funding Statement section of the online submission form. Please remove any funding-related text from the manuscript.

- We have removed the related content from the manuscript.

6.We noted in your submission details that a portion of your manuscript may have been presented or published elsewhere.

"No, only a basic version has been posted on arXiv as a preprint; this manuscript has undergone significant revisions."
Please clarify whether this [conference proceeding or publication] was peer-reviewed and formally published. If this work was previously peer-reviewed and published, in the cover letter please provide the reason that this work does not constitute dual publication and should be included in the current manuscript.
- The basic version is available on arXiv (https://arxiv.org/abs/2401.03171). This manuscript is only a preprint; it has not undergone peer review and has not been formally published in any conference or journal. Therefore, it does not constitute a dual publication.

7.If the reviewer comments include a recommendation to cite specific previously published works, please review and evaluate these publications to determine whether they are relevant and should be cited. There is no requirement to cite these works unless the editor has indicated otherwise.
- No additional references were requested by the reviewers.

Response Letter for Reviewers

Response for Reviewer#1

1.The most important issue is what I wonder if the conceptual idea would work in clinical diagnosis. First of all, depression appears in more than 90% of adolescent in many countries, it's pretty easy to diagnose using current metrics. The difficulty nowadays is more of precaution before diagnosis rather than diagnose the symptoms. Maybe the author should address your major finding/aims from this perspective. Secondly, I highly doubt this paper can be called diagnosis tool. It's very much against how diagnosis from individual perspective to macro level as in this paper. Census survey can only be used for prediction for future or verifying the past. How could it be used to diagnosis if certain kids have depression now? Maybe the most of it can be described as "the depression status of certain areas". This point is in line with my first point that the method in the paper might not be able to be supported by the data. It's indeed a challenge though.

- We thank the reviewers for their professional and constructive comments. We have revised the wording in the manuscript to clarify that our contribution is to use predictive algorithms to assess the risk of adolescent depression, rather than to directly support clinical diagnosis.

2.The method of this paper is super difficult to follow. As a reader, I would believe for other readers too, I found the method sections appear here and there in the many parts of the paper. Especially in the result, it should just show results instead of involving more method.

- We have substantially revised the structure of the manuscript. Specifically, we moved details such as the experimental setup to the final part of the Methods section, and removed method-related details that were incorrectly placed in the Results section. Please refer to the tracked-changes version (Lines 294–336 and 381–392) for details.

3.The representation of the results in the study is quite confusing for me. All the visualizations and tables need to be unified as publishable standard, for now, the font, the color, the color palettes, the format are not ready. More importantly, the structure of how the results are shown should be reconsidered. I would suggest either follow the clinical standard to show before and after to show the results or follow the machine learning method to show the model results. Mixing together without a clear storytelling might not be the best option.

We unified the fonts used in all figures and tables and updated the figure style accordingly. Details can be found in the tracked-changes version, where the updated figures are highlighted in blue boxes. We also standardized the line styles in the tables.

Response for Reviewer#2

1. Conceptual Clarification: Diagnosis vs. Risk Prediction

The manuscript frequently refers to the proposed model as performing “diagnosis,” including in the title and Introduction. However, given that the model is trained on census-derived, largely self- or parent-reported variables and is not validated against clinician-administered diagnostic interviews or standardized diagnostic assessments, its function aligns more closely with risk prediction or screening support rather than clinical diagnosis. From a mental health perspective, this distinction is important to ensure conceptual accuracy and avoid overinterpretation

Suggestion:

Consider revising terminology throughout the manuscript to reflect risk prediction, risk stratification, or screening support, rather than diagnosis. This clarification would better align the language with the study design and strengthen the scientific rigor of the work.

- We thank the reviewers for their professional and constructive comments. We have revised the wording in the manuscript to clarify that our contribution is to use predictive algorithms to assess the risk of adolescent depression, rather than to directly support clinical diagnosis.

2. Intended Level of Application and Clinical Context

While the manuscript suggests that the system may be accessible to ordinary users and potentially deployed in clinical settings, the intended level of application is not clearly specified. Based on the census-based data source and absence of clinical validation, the model appears more suitable for population-level or public health screening contexts than for individual diagnostic decision-making.

Suggestion:

Explicitly state the intended use context of the model and clarify its role within existing healthcare or public health workflows to improve translational clarity.

- We now explicitly state the intended use context in a new Intended Application subsection. The model is positioned as a public health/school screening tool to flag potentially at-risk adolescents for follow-up evaluation, not as a stand-alone diagnostic system, given the census-based data source and lack of clinical validation.

3. Interpretation of Performance Metrics in Clinical Context

The reported performance metrics demonstrate strong technical performance, and the authors appropriately discuss issues related to class imbalance. However, from a clinical standpoint, high predictive metrics do not necessarily translate into diagnostic utility or improved clinical decision-making, particularly in the absence of evaluation of downstream consequences.

Suggestion:

In the Discussion, consider contextualizing performance metrics more explicitly within a clinical or public health decision-making framework, emphasizing that the reported

metrics demonstrate technical validity rather than confirmed clinical effectiveness.

- We revised the Discussion to clarify that the reported metrics indicate predictive accuracy for depression risk screening/risk assessment, not clinical diagnostic accuracy or confirmed clinical utility.

4. Interpretability and Clinical Actionability of SHAP Analyses

The SHAP-based interpretability analyses represent a methodological strength and provide transparency regarding feature contributions. Nevertheless, interpretability at the model level does not automatically imply clinical actionability, and the manuscript does not assess whether these explanations would meaningfully inform clinical judgment or intervention planning.

Suggestion:

Acknowledge this distinction in the Discussion and clarify that the interpretability analyses primarily support model transparency and hypothesis generation, rather than direct clinical decision-making at this stage.

- We now explicitly state that the interpretability of the model including the SHAP analyses only support model transparency and hypotheses generation, but do not validate the model's ability to inform real-world clinical decisions, at the end of the 'Interpretability results' part in the Results, and the 'Interpretation of Model Interpretability' part in the Discussion.

5. Generalizability and Cultural Context

The study appropriately notes that the data are derived from U.S. census sources; however, several key predictors are highly sensitive to cultural, socioeconomic, and health system contexts. This limits the immediate generalizability of the findings to other populations.

Suggestion:

Expand the Discussion on cultural and contextual variability and emphasize the need for external validation in diverse populations before broader application.

- In the original version, we acknowledged the limitation regarding the U.S. census data in the 'Limitations' section in the Discussion, clarifying that the findings may not represent individuals from all over the world. To further emphasize that, we now re-state the fact as well as the need for clinical experiments before application by the end of this part.

6. Conclusions and Strength of Claims

The Conclusion highlights the potential contribution of the proposed system to adolescent depression identification and prevention. However, some statements may exceed what can be directly supported by the observational and predictive nature of the study.

Suggestion:

Adopt more conservative language in the Conclusion that reflects the study's role in advancing risk prediction and screening research, while clearly identifying clinical implementation and outcome evaluation as future steps.

- We revised the Conclusion to clarify the role of our system as an auxiliary diagnosis tool to assist mental disorder prevention or detection, and explicitly point out the need for further clinical implementation and outcome evaluation.

7. Ethical Considerations and Safeguards

Given the sensitivity of adolescent mental health data and the potential consequences of algorithmic risk labeling, ethical considerations deserve explicit attention.

Suggestion:

Include a brief statement in the Discussion or Conclusion noting that any real-world implementation should occur within structured clinical oversight frameworks, with clear referral pathways and safeguards to minimize misclassification, stigma, and unintended harm.

-We have added a brief "Ethical considerations and safeguards" statement noting that the data are publicly available, deidentified, and approved for research use, and that any real-world deployment should occur under clinical oversight with clear referral pathways and safeguards to reduce misclassification, stigma, and unintended harm.
