# Supplementary material for: Census-based adolescent depression risk assessment: A novel method and reflections
Source: PLoS One. 2026 Sep 15;21(9):e0357661. doi: 10.1371/journal.pone.0357661 (PMC13577525; doi:10.1371/journal.pone.0357661)
Supplement: Supplementary file 3 [file pone.0357661.s004.pdf]

Review Response Letter

Reviewer #3

1. Add calibration metrics (Brier score, ECE) to ensure accurate risk estimation and strengthen public-health applicability.

- We thanks for the suggestion. We have incorporated additional brier score and Expected Calibration Error (ECE) experiments. The setup is now described in the Experimental Setup section, and the results are presented in a new subsection, Calibration of risk predictions, placed just before the interpretability analysis in the Results section.

2. Minor methodological and presentational refinements are needed, but they do not compromise the paper's core contributions.

- Thanks for the positive assessment. We have revised the manuscript by incorporating the suggested methodological and presentational refinements. In particular, corresponding descriptions in the experimental setup section have been clarified and supplemented to improve the completeness and reproducibility of the study.

3. The paper requires a dedicated reproducibility section detailing hardware, runtime, software, and AutoML framework to ensure transparency.

- We have added a computational environment section describing the hardware configuration, software versions, AutoML framework, and experimental settings to improve the reproducibility and transparency of our study.

4. Specify dataset version and download date to improve reproducibility.

- The Data resource subsection has been revised, and additional information regarding the dataset source, survey years, and acquisition date has been included to improve reproducibility and transparency.

Reviewer #4

1. The framing of the work as risk prediction rather than diagnosis is appropriate and resolves conceptual concerns. Make sure this distinction is consistently maintained throughout the manuscript.

- Thank you for the suggestion. We have revised the manuscript to consistently describe AADA as a risk prediction and screening-support framework rather than a diagnostic system. Terminology related to risk assessment, screening, and clinical interpretation has been refined throughout the manuscript.

2. Provide clearer details on data partitioning, synthetic data generation and how class imbalance was handled. Without a clear description, readers may question whether the reported performance is truly generalisable.

- We have clarified the data partitioning and class-imbalance handling in the Experimental Setup section. The revised manuscript now specifies that both test sets were reserved for evaluation, while M-SMOTE, ENN, and CensusFilter-based processing were restricted to the training data.

3. Ensure that figures and tables conform to publication standards (consistent fonts, colours, and legibility). Remove any remnants of track changes.

- We have reviewed all figures and tables and adjusted their formatting to improve consistency and readability. Some figure elements were resized to maintain appropriate layout and legibility. We have also provided both clean and tracked-changes versions, with all track-change marks removed from the clean manuscript.

4. Add a link to the GitHub repository or upload the code as supplementary information to enhance reproducibility.

- In the previous version, we already included the GitHub link in the footnote below the abstract on the first page, and this link has been retained in the revised manuscript. Additionally, we have updated the code repository to facilitate easier reproduction.

5. Continue to refine the language to correct grammatical errors and avoid ambiguous terms. A copy - edit will improve readability.

We have carefully proofread the manuscript and refined the language throughout the text. Grammatical issues were corrected, and ambiguous expressions were revised to improve clarity, consistency, and readability.
