# Supplementary material for: Census-based adolescent depression risk assessment: A novel method and reflections
Source: PLoS One. 2026 Sep 15;21(9):e0357661. doi: 10.1371/journal.pone.0357661 (PMC13577525; doi:10.1371/journal.pone.0357661)
Supplement: Peer review history

# Peer review: Decision Letter 0

**DOI:** 10.1371/journal.pone.0357661.r001

**Authors:** Leona Cilar Budler

26 Dec 2025

Dear Dr. Zhou,

Thank you for submitting your manuscript to PLOS ONE. After careful consideration, we feel that it has merit but does not fully meet PLOS ONE’s publication criteria as it currently stands. Therefore, we invite you to submit a revised version of the manuscript that addresses the points raised during the review process.

plosone@plos.org. When you're ready to submit your revision, log on to https://www.editorialmanager.com/pone/ and select the 'Submissions Needing Revision' folder to locate your manuscript file.

- A letter that responds to each point raised by the academic editor and reviewer(s). You should upload this letter as a separate file labeled 'Response to Reviewers'.
- A marked-up copy of your manuscript that highlights changes made to the original version. You should upload this as a separate file labeled 'Revised Manuscript with Track Changes'.
- An unmarked version of your revised paper without tracked changes. You should upload this as a separate file labeled 'Manuscript'.

If you would like to make changes to your financial disclosure, please include your updated statement in your cover letter. Guidelines for resubmitting your figure files are available below the reviewer comments at the end of this letter.

If applicable, we recommend that you deposit your laboratory protocols in protocols.io to enhance the reproducibility of your results. Protocols.io assigns your protocol its own identifier (DOI) so that it can be cited independently in the future. For instructions see: https://journals.plos.org/plosone/s/submission-guidelines#loc-laboratory-protocols. Additionally, PLOS ONE offers an option for publishing peer-reviewed Lab Protocol articles, which describe protocols hosted on protocols.io. Read more information on sharing protocols at https://plos.org/protocols?utm_medium=editorial-email&utm_source=authorletters&utm_campaign=protocols.

We look forward to receiving your revised manuscript.

Kind regards,

Leona Cilar Budler

Academic Editor

PLOS One

**Journal Requirements:**

When submitting your revision, we need you to address these additional requirements.

1. Please ensure that your manuscript meets PLOS ONE's style requirements, including those for file naming. The PLOS ONE style templates can be found at

https://journals.plos.org/plosone/s/file?id=wjVg/PLOSOne_formatting_sample_main_body.pdf and

https://journals.plos.org/plosone/s/file?id=ba62/PLOSOne_formatting_sample_title_authors_affiliations.pdf

2. Please note that PLOS ONE has specific guidelines on code sharing for submissions in which author-generated code underpins the findings in the manuscript. In these cases, we expect all author-generated code to be made available without restrictions upon publication of the work. Please review our guidelines at https://journals.plos.org/plosone/s/materials-and-software-sharing#loc-sharing-code and ensure that your code is shared in a way that follows best practice and facilitates reproducibility and reuse.

3. We note that the grant information you provided in the ‘Funding Information’ and ‘Financial Disclosure’ sections do not match.

When you resubmit, please ensure that you provide the correct grant numbers for the awards you received for your study in the ‘Funding Information’ section.

4. Thank you for stating the following financial disclosure:

“This research was funded by Research topic on teaching reform in higher education 551

institutions in Jiangxi Province in 2022 grant number: JXJG-22-16-15.”

Please state what role the funders took in the study.  If the funders had no role, please state: "The funders had no role in study design, data collection and analysis, decision to publish, or preparation of the manuscript."

If this statement is not correct you must amend it as needed.

Please include this amended Role of Funder statement in your cover letter; we will change the online submission form on your behalf.

5. Please note that funding information should not appear in any section or other areas of your manuscript. We will only publish funding information present in the Funding Statement section of the online submission form. Please remove any funding-related text from the manuscript.

6. We noted in your submission details that a portion of your manuscript may have been presented or published elsewhere.

“No, only a basic version has been posted on arXiv as a preprint; this manuscript has undergone significant revisions.”

Please clarify whether this [conference proceeding or publication] was peer-reviewed and formally published. If this work was previously peer-reviewed and published, in the cover letter please provide the reason that this work does not constitute dual publication and should be included in the current manuscript.

7. If the reviewer comments include a recommendation to cite specific previously published works, please review and evaluate these publications to determine whether they are relevant and should be cited. There is no requirement to cite these works unless the editor has indicated otherwise.

**Additional Editor Comments:**

There are some major issues listed by reviewers. Read all comments and suggestions to improve paper quality. Also, revise methodological issues and justify study need.

[Note: HTML markup is below. Please do not edit.]

Reviewers' comments:

Reviewer's Responses to Questions

**Comments to the Author**

1. Is the manuscript technically sound, and do the data support the conclusions?

Reviewer #1: Partly

Reviewer #2: Yes

**********

2. Has the statistical analysis been performed appropriately and rigorously?

Reviewer #1: Yes

Reviewer #2: Yes

**********

3. Have the authors made all data underlying the findings in their manuscript fully available?

The PLOS Data policy

Reviewer #1: Yes

Reviewer #2: Yes

**********

4. Is the manuscript presented in an intelligible fashion and written in standard English?

Reviewer #1: Yes

Reviewer #2: Yes

**********

Reviewer #1: This paper uses U.S. census survey data (NSCH 2020–2022) to build a machine learning model for predicting adolescent depression. However, there are some major issues I would like to discuss with the authors.

1. The most important issue is what I wonder if the conceptual idea would work in clinical diagnosis. First of all, depression appears in more than 90% of adolescent in many countries, it's pretty easy to diagnose using current metrics. The difficulty nowadays is more of precaution before diagnosis rather than diagnose the symptoms. Maybe the author should address your major finding/aims from this perspective. Secondly, I highly doubt this paper can be called diagnosis tool. It's very much against how diagnosis from individual perspective to macro level as in this paper. Census survey can only be used for prediction for future or verifying the past. How could it be used to diagnosis if certain kids have depression now? Maybe the most of it can be described as "the depression status of certain areas". This point is in line with my first point that the method in the paper might not be able to be supported by the data. It's indeed a challenge though.

2. The method of this paper is super difficult to follow. As a reader, I would believe for other readers too, I found the method sections appear here and there in the many parts of the paper. Especially in the result, it should just show results instead of involving more method.

3. The representation of the results in the study is quite confusing for me. All the visualizations and tables need to be unified as publishable standard, for now, the font, the color, the color palettes, the format are not ready. More importantly, the structure of how the results are shown should be reconsidered. I would suggest either follow the clinical standard to show before and after to show the results or follow the machine learning method to show the model results. Mixing together without a clear storytelling might not be the best option.

Reviewer #2: Reviewer Comments

This manuscript presents a technically rigorous machine learning framework applied to a large, nationally representative census dataset to address adolescent depression risk. Key strengths include the use of high-quality population-level data, a well-described and methodologically sound approach to handling class imbalance, and the integration of adaptive AutoML and ensemble strategies that enhance robustness and reproducibility compared with prior static models in the field. In addition, the use of SHAP-based explainability aligns with current best practices in explainable artificial intelligence, and the identified predictors are clinically and epidemiologically plausible. Overall, the study makes a solid and relevant contribution to the literature on AI-based risk prediction in adolescent mental health, particularly from a population and public health perspective.

Specific Comments and Suggestions

1. Conceptual Clarification: Diagnosis vs. Risk Prediction

The manuscript frequently refers to the proposed model as performing “diagnosis,” including in the title and Introduction. However, given that the model is trained on census-derived, largely self- or parent-reported variables and is not validated against clinician-administered diagnostic interviews or standardized diagnostic assessments, its function aligns more closely with risk prediction or screening support rather than clinical diagnosis. From a mental health perspective, this distinction is important to ensure conceptual accuracy and avoid overinterpretation

Suggestion:

Consider revising terminology throughout the manuscript to reflect risk prediction, risk stratification, or screening support, rather than diagnosis. This clarification would better align the language with the study design and strengthen the scientific rigor of the work.

2. Intended Level of Application and Clinical Context

While the manuscript suggests that the system may be accessible to ordinary users and potentially deployed in clinical settings, the intended level of application is not clearly specified. Based on the census-based data source and absence of clinical validation, the model appears more suitable for population-level or public health screening contexts than for individual diagnostic decision-making.

Suggestion:

Explicitly state the intended use context of the model and clarify its role within existing healthcare or public health workflows to improve translational clarity.

3. Interpretation of Performance Metrics in Clinical Context

The reported performance metrics demonstrate strong technical performance, and the authors appropriately discuss issues related to class imbalance. However, from a clinical standpoint, high predictive metrics do not necessarily translate into diagnostic utility or improved clinical decision-making, particularly in the absence of evaluation of downstream consequences.

Suggestion:

In the Discussion, consider contextualizing performance metrics more explicitly within a clinical or public health decision-making framework, emphasizing that the reported metrics demonstrate technical validity rather than confirmed clinical effectiveness.

4. Interpretability and Clinical Actionability of SHAP Analyses

The SHAP-based interpretability analyses represent a methodological strength and provide transparency regarding feature contributions. Nevertheless, interpretability at the model level does not automatically imply clinical actionability, and the manuscript does not assess whether these explanations would meaningfully inform clinical judgment or intervention planning.

Suggestion:

Acknowledge this distinction in the Discussion and clarify that the interpretability analyses primarily support model transparency and hypothesis generation, rather than direct clinical decision-making at this stage.

5. Generalizability and Cultural Context

The study appropriately notes that the data are derived from U.S. census sources; however, several key predictors are highly sensitive to cultural, socioeconomic, and health system contexts. This limits the immediate generalizability of the findings to other populations.

Suggestion:

Expand the Discussion on cultural and contextual variability and emphasize the need for external validation in diverse populations before broader application.

6. Conclusions and Strength of Claims

The Conclusion highlights the potential contribution of the proposed system to adolescent depression identification and prevention. However, some statements may exceed what can be directly supported by the observational and predictive nature of the study.

Suggestion:

Adopt more conservative language in the Conclusion that reflects the study’s role in advancing risk prediction and screening research, while clearly identifying clinical implementation and outcome evaluation as future steps.

7. Ethical Considerations and Safeguards

Given the sensitivity of adolescent mental health data and the potential consequences of algorithmic risk labeling, ethical considerations deserve explicit attention.

Suggestion:

Include a brief statement in the Discussion or Conclusion noting that any real-world implementation should occur within structured clinical oversight frameworks, with clear referral pathways and safeguards to minimize misclassification, stigma, and unintended harm.

**********

what does this mean?). If published, this will include your full peer review and any attached files.

If you choose “no”, your identity will remain anonymous but your review may still be made public.

**Do you want your identity to be public for this peer review?**  For information about this choice, including consent withdrawal, please see our Privacy Policy

Reviewer #1: No

Reviewer #2: No

**********

[NOTE: If reviewer comments were submitted as an attachment file, they will be attached to this email and accessible via the submission site. Please log into your account, locate the manuscript record, and check for the action link "View Attachments". If this link does not appear, there are no attachment files.]

To ensure your figures meet our technical requirements, please review our figure guidelines: https://journals.plos.org/plosone/s/figures

You may also use PLOS’s free figure tool, NAAS, to help you prepare publication quality figures: https://journals.plos.org/plosone/s/figures#loc-tools-for-figure-preparation.

NAAS will assess whether your figures meet our technical requirements by comparing each figure against our figure specifications.

# Author comment: Author response to Decision Letter 1

**DOI:** 10.1371/journal.pone.0357661.r002

18 Apr 2026

Response for Reviewer#1

1.The most important issue is what I wonder if the conceptual idea would work in clinical diagnosis. First of all, depression appears in more than 90% of adolescent in many countries, it's pretty easy to diagnose using current metrics. The difficulty nowadays is more of precaution before diagnosis rather than diagnose the symptoms. Maybe the author should address your major finding/aims from this perspective. Secondly, I highly doubt this paper can be called diagnosis tool. It's very much against how diagnosis from individual perspective to macro level as in this paper. Census survey can only be used for prediction for future or verifying the past. How could it be used to diagnosis if certain kids have depression now? Maybe the most of it can be described as "the depression status of certain areas". This point is in line with my first point that the method in the paper might not be able to be supported by the data. It's indeed a challenge though.

- We thank the reviewers for their professional and constructive comments. We have revised the wording in the manuscript to clarify that our contribution is to use predictive algorithms to assess the risk of adolescent depression, rather than to directly support clinical diagnosis.

2.The method of this paper is super difficult to follow. As a reader, I would believe for other readers too, I found the method sections appear here and there in the many parts of the paper. Especially in the result, it should just show results instead of involving more method.

- We have substantially revised the structure of the manuscript. Specifically, we moved details such as the experimental setup to the final part of the Methods section, and removed method-related details that were incorrectly placed in the Results section. Please refer to the tracked-changes version (Lines 294–336 and 381–392) for details.

3.The representation of the results in the study is quite confusing for me. All the visualizations and tables need to be unified as publishable standard, for now, the font, the color, the color palettes, the format are not ready. More importantly, the structure of how the results are shown should be reconsidered. I would suggest either follow the clinical standard to show before and after to show the results or follow the machine learning method to show the model results. Mixing together without a clear storytelling might not be the best option.

We unified the fonts used in all figures and tables and updated the figure style accordingly. Details can be found in the tracked-changes version, where the updated figures are highlighted in blue boxes. We also standardized the line styles in the tables.

Response for Reviewer#2

1. Conceptual Clarification: Diagnosis vs. Risk Prediction

The manuscript frequently refers to the proposed model as performing “diagnosis,” including in the title and Introduction. However, given that the model is trained on census-derived, largely self- or parent-reported variables and is not validated against clinician-administered diagnostic interviews or standardized diagnostic assessments, its function aligns more closely with risk prediction or screening support rather than clinical diagnosis. From a mental health perspective, this distinction is important to ensure conceptual accuracy and avoid overinterpretation

Suggestion:

Consider revising terminology throughout the manuscript to reflect risk prediction, risk stratification, or screening support, rather than diagnosis. This clarification would better align the language with the study design and strengthen the scientific rigor of the work.

- We thank the reviewers for their professional and constructive comments. We have revised the wording in the manuscript to clarify that our contribution is to use predictive algorithms to assess the risk of adolescent depression, rather than to directly support clinical diagnosis.

2. Intended Level of Application and Clinical Context

While the manuscript suggests that the system may be accessible to ordinary users and potentially deployed in clinical settings, the intended level of application is not clearly specified. Based on the census-based data source and absence of clinical validation, the model appears more suitable for population-level or public health screening contexts than for individual diagnostic decision-making.

Suggestion:

Explicitly state the intended use context of the model and clarify its role within existing healthcare or public health workflows to improve translational clarity.

- We now explicitly state the intended use context in a new Intended Application subsection. The model is positioned as a public health/school screening tool to flag potentially at-risk adolescents for follow-up evaluation, not as a stand-alone diagnostic system, given the census-based data source and lack of clinical validation.

3. Interpretation of Performance Metrics in Clinical Context

The reported performance metrics demonstrate strong technical performance, and the authors appropriately discuss issues related to class imbalance. However, from a clinical standpoint, high predictive metrics do not necessarily translate into diagnostic utility or improved clinical decision-making, particularly in the absence of evaluation of downstream consequences.

Suggestion:

In the Discussion, consider contextualizing performance metrics more explicitly within a clinical or public health decision-making framework, emphasizing that the reported metrics demonstrate technical validity rather than confirmed clinical effectiveness.

- We revised the Discussion to clarify that the reported metrics indicate predictive accuracy for depression risk screening/risk assessment, not clinical diagnostic accuracy or confirmed clinical utility.

4. Interpretability and Clinical Actionability of SHAP Analyses

The SHAP-based interpretability analyses represent a methodological strength and provide transparency regarding feature contributions. Nevertheless, interpretability at the model level does not automatically imply clinical actionability, and the manuscript does not assess whether these explanations would meaningfully inform clinical judgment or intervention planning.

Suggestion:

Acknowledge this distinction in the Discussion and clarify that the interpretability analyses primarily support model transparency and hypothesis generation, rather than direct clinical decision-making at this stage.

- We now explicitly state that the interpretability of the model including the SHAP analyses only support model transparency and hypotheses generation, but do not validate the model’s ability to inform real-world clinical decisions, at the end of the ‘Interpretability results’ part in the Results, and the ‘Interpretation of Model Interpretability’ part in the Discussion.

5. Generalizability and Cultural Context

The study appropriately notes that the data are derived from U.S. census sources; however, several key predictors are highly sensitive to cultural, socioeconomic, and health system contexts. This limits the immediate generalizability of the findings to other populations.

Suggestion:

Expand the Discussion on cultural and contextual variability and emphasize the need for external validation in diverse populations before broader application.

- In the original version, we acknowledged the limitation regarding the U.S. census data in the 'Limitations' section in the Discussion, clarifying that the findings may not represent individuals from all over the world. To further emphasize that, we now re-state the fact as well as the need for clinical experiments before application by the end of this part.

6. Conclusions and Strength of Claims

The Conclusion highlights the potential contribution of the proposed system to adolescent depression identification and prevention. However, some statements may exceed what can be directly supported by the observational and predictive nature of the study.

Suggestion:

Adopt more conservative language in the Conclusion that reflects the study’s role in advancing risk prediction and screening research, while clearly identifying clinical implementation and outcome evaluation as future steps.

- We revised the Conclusion to clarify the role of our system as an auxiliary diagnosis tool to assist mental disorder prevention or detection, and explicitly point out the need for further clinical implementation and outcome evaluation.

7. Ethical Considerations and Safeguards

Given the sensitivity of adolescent mental health data and the potential consequences of algorithmic risk labeling, ethical considerations deserve explicit attention.

Suggestion:

Include a brief statement in the Discussion or Conclusion noting that any real-world implementation should occur within structured clinical oversight frameworks, with clear referral pathways and safeguards to minimize misclassification, stigma, and unintended harm.

-We have added a brief “Ethical considerations and safeguards” statement noting that the data are publicly available, deidentified, and approved for research use, and that any real-world deployment should occur under clinical oversight with clear referral pathways and safeguards to reduce misclassification, stigma, and unintended harm.

Attachment Submitted filename: Response Letter PLOSONE r1.pdf

# Peer review: Decision Letter 1

**DOI:** 10.1371/journal.pone.0357661.r003

**Authors:** Mohamed Baklola

3 Jun 2026

Dear Dr. Li,

Thank you for submitting your manuscript to PLOS ONE. After careful consideration, we feel that it has merit but does not fully meet PLOS ONE’s publication criteria as it currently stands. Therefore, we invite you to submit a revised version of the manuscript that addresses the points raised during the review process.

Please submit your revised manuscript by Jul 18 2026 11:59PM. If you will need more time than this to complete your revisions, please reply to this message or contact the journal office at plosone@plos.org. When you're ready to submit your revision, log on to https://www.editorialmanager.com/pone/ and select the 'Submissions Needing Revision' folder to locate your manuscript file.

- A letter that responds to each point raised by the academic editor and reviewer(s). You should upload this letter as a separate file labeled 'Response to Reviewers'.
- A marked-up copy of your manuscript that highlights changes made to the original version. You should upload this as a separate file labeled 'Revised Manuscript with Track Changes'.
- An unmarked version of your revised paper without tracked changes. You should upload this as a separate file labeled 'Manuscript'.

If you would like to make changes to your financial disclosure, please include your updated statement in your cover letter. Guidelines for resubmitting your figure files are available below the reviewer comments at the end of this letter.

If applicable, we recommend that you deposit your laboratory protocols in protocols.io to enhance the reproducibility of your results. Protocols.io assigns your protocol its own identifier (DOI) so that it can be cited independently in the future. For instructions see: https://journals.plos.org/plosone/s/submission-guidelines#loc-laboratory-protocols. Additionally, PLOS ONE offers an option for publishing peer-reviewed Lab Protocol articles, which describe protocols hosted on protocols.io. Read more information on sharing protocols at https://plos.org/protocols?utm_medium=editorial-email&utm_source=authorletters&utm_campaign=protocols.

As the corresponding author, your ORCID iD is verified in the submission system and will appear in the published article. PLOS supports the use of ORCID, and we encourage all coauthors to register for an ORCID iD and use it as well. Please encourage your coauthors to verify their ORCID iD within the submission system before final acceptance, as unverified ORCID iDs will not appear in the published article. *Only*  the individual author can complete the verification step; PLOS staff *cannot*  verify ORCID iDs on behalf of authors.

We look forward to receiving your revised manuscript.

Kind regards,

Mohamed Baklola

Academic Editor

PLOS One

Journal Requirements:

If the reviewer comments include a recommendation to cite specific previously published works, please review and evaluate these publications to determine whether they are relevant and should be cited. There is no requirement to cite these works unless the editor has indicated otherwise.

Please review your reference list to ensure that it is complete and correct. If you have cited papers that have been retracted, please include the rationale for doing so in the manuscript text, or remove these references and replace them with relevant current references. Any changes to the reference list should be mentioned in the rebuttal letter that accompanies your revised manuscript. If you need to cite a retracted article, indicate the article’s retracted status in the References list and also include a citation and full reference for the retraction notice.

Additional Editor Comments:

The remaining issues are relatively minor and mainly concern methodological transparency, reproducibility, and presentation. Please address the following points carefully in your next revision:

1) Since the work focuses on risk assessment, please consider including calibration-related evaluation metrics and/or visualizations (e.g., calibration plots, reliability curves, Brier score, or expected calibration error). This would strengthen the public-health applicability of the model because strong discrimination performance alone may not guarantee well-calibrated risk estimates.

2) Please provide clearer details regarding:

- data partitioning procedures,

- handling of class imbalance,

- synthetic data generation methods (if applicable), and

- safeguards against preprocessing leakage.

3) Improve reproducibility reporting by adding a dedicated subsection describing:

- software packages and versions,

- hardware/computational environment,

- runtime information,

- AutoML framework details (if used), and

- the exact dataset version and download/access date.

4) Please add a link to the code repository (e.g., GitHub) or provide code as supplementary material to enhance computational reproducibility.

5) Expand the discussion of ethical and societal implications of AI-based risk assessment systems, including potential biases, limitations, and the importance of appropriate oversight in real-world deployment.

6) Ensure that all figures and tables conform to journal presentation standards, including consistent formatting, readable labels/fonts, and removal of any remaining track-change artifacts.

7) Please continue refining the language and grammar throughout the manuscript to improve clarity and readability.

[Note: HTML markup is below. Please do not edit.]

Reviewers' comments:

Reviewer's Responses to Questions

**Comments to the Author**

Reviewer #3: All comments have been addressed

Reviewer #4: (No Response)

**********

2. Is the manuscript technically sound, and do the data support the conclusions?

Reviewer #3: Yes

Reviewer #4: Partly

**********

3. Has the statistical analysis been performed appropriately and rigorously?

Reviewer #3: Yes

Reviewer #4: I Don't Know

**********

4. Have the authors made all data underlying the findings in their manuscript fully available?

The PLOS Data policy

Reviewer #3: Yes

Reviewer #4: Yes

**********

5. Is the manuscript presented in an intelligible fashion and written in standard English?

Reviewer #3: Yes

Reviewer #4: Yes

**********

Reviewer #3: Since the paper proposes risk assessment rather than classification alone, calibration matters. Consider adding calibration plots, Brier score, expected calibration error (ECE), or reliability curves. Because a model with good AUC may still produce poorly calibrated risk estimates. This would substantially strengthen the public-health applicability argument.

Some methodological and presentation issues remain that could further strengthen the manuscript (e.g., clarification of survey weighting, preprocessing leakage prevention, and statistical comparison reporting), but these are relatively minor and do not fundamentally undermine the overall contribution of the work.

For a computational paper, several reproducibility details are missing hardware, runtime, software versions, and AutoML framework used. A dedicated reproducibility paragraph would improve transparency greatly.

One minor data improvement would be for the authors to provide the exact dataset version/download date to improve computational reproducibility. However, these issues do not constitute a violation of the PLOS Data policy.

Overall, I believe the manuscript is now acceptable for publication after very minor editorial refinement.

Reviewer #4: - The framing of the work as risk prediction rather than diagnosis is appropriate and resolves conceptual concerns. Make sure this distinction is consistently maintained throughout the manuscript.

- Provide clearer details on data partitioning, synthetic data generation and how class imbalance was handled. Without a clear description, readers may question whether the reported performance is truly generalisable.

- Ensure that figures and tables conform to publication standards (consistent fonts, colours, and legibility). Remove any remnants of track changes.

- Add a link to the GitHub repository or upload the code as supplementary information to enhance reproducibility.

- Discuss ethical and societal implications of deploying AI‑based risk assessments, including potential biases and the need for oversight.

- Continue to refine the language to correct grammatical errors and avoid ambiguous terms. A copy‑edit will improve readability.

**********

what does this mean?). If published, this will include your full peer review and any attached files.

If you choose “no”, your identity will remain anonymous but your review may still be made public.

**Do you want your identity to be public for this peer review?**  For information about this choice, including consent withdrawal, please see our Privacy Policy

Reviewer #3: No

Reviewer #4: **Yes:** Jackson Shawa

**********

[NOTE: If reviewer comments were submitted as an attachment file, they will be attached to this email and accessible via the submission site. Please log into your account, locate the manuscript record, and check for the action link "View Attachments". If this link does not appear, there are no attachment files.]

To ensure your figures meet our technical requirements, please review our figure guidelines: https://journals.plos.org/plosone/s/figures

You may also use PLOS’s free figure tool, NAAS, to help you prepare publication quality figures: https://journals.plos.org/plosone/s/figures#loc-tools-for-figure-preparation.

NAAS will assess whether your figures meet our technical requirements by comparing each figure against our figure specifications.

# Author comment: Author response to Decision Letter 2

**DOI:** 10.1371/journal.pone.0357661.r004

18 Aug 2026

Reviewer #3

1. Add calibration metrics (Brier score, ECE) to ensure accurate risk estimation and strengthen public-health applicability.

- We thanks for the suggestion. We have incorporated additional brier score and Expected Calibration Error (ECE) experiments. The setup is now described in the Experimental Setup section, and the results are presented in a new subsection, Calibration of risk predictions, placed just before the interpretability analysis in the Results section.

2. Minor methodological and presentational refinements are needed, but they do not compromise the paper's core contributions.

- Thanks for the positive assessment. We have revised the manuscript by incorporating the suggested methodological and presentational refinements. In particular, corresponding descriptions in the experimental setup section have been clarified and supplemented to improve the completeness and reproducibility of the study.

3. The paper requires a dedicated reproducibility section detailing hardware, runtime, software, and AutoML framework to ensure transparency.

- We have added a computational environment section describing the hardware configuration, software versions, AutoML framework, and experimental settings to improve the reproducibility and transparency of our study.

4. Specify dataset version and download date to improve reproducibility.

- The Data resource subsection has been revised, and additional information regarding the dataset source, survey years, and acquisition date has been included to improve reproducibility and transparency.

Reviewer #4

1. The framing of the work as risk prediction rather than diagnosis is appropriate and resolves conceptual concerns. Make sure this distinction is consistently maintained throughout the manuscript.

- Thank you for the suggestion. We have revised the manuscript to consistently describe AADA as a risk prediction and screening-support framework rather than a diagnostic system. Terminology related to risk assessment, screening, and clinical interpretation has been refined throughout the manuscript.

2. Provide clearer details on data partitioning, synthetic data generation and how class imbalance was handled. Without a clear description, readers may question whether the reported performance is truly generalisable.

- We have clarified the data partitioning and class-imbalance handling in the Experimental Setup section. The revised manuscript now specifies that both test sets were reserved for evaluation, while M-SMOTE, ENN, and CensusFilter-based processing were restricted to the training data.

3. Ensure that figures and tables conform to publication standards (consistent fonts, colours, and legibility). Remove any remnants of track changes.

- We have reviewed all figures and tables and adjusted their formatting to improve consistency and readability. Some figure elements were resized to maintain appropriate layout and legibility. We have also provided both clean and tracked-changes versions, with all track-change marks removed from the clean manuscript.

4. Add a link to the GitHub repository or upload the code as supplementary information to enhance reproducibility.

- In the previous version, we already included the GitHub link in the footnote below the abstract on the first page, and this link has been retained in the revised manuscript. Additionally, we have updated the code repository to facilitate easier reproduction.

5. Continue to refine the language to correct grammatical errors and avoid ambiguous terms. A copy‑edit will improve readability.

We have carefully proofread the manuscript and refined the language throughout the text. Grammatical issues were corrected, and ambiguous expressions were revised to improve clarity, consistency, and readability.

Attachment Submitted filename: Review_Response_Letter_R2.pdf

# Editor report: Decision Letter 2

**DOI:** 10.1371/journal.pone.0357661.r005

**Authors:** Mohamed Baklola

20 Aug 2026

Census-Based Adolescent Depression Risk Assessment: A Novel Method and Reflections

PONE-D-25-28656R2

Dear Dr. Li,

We’re pleased to inform you that your manuscript has been judged scientifically suitable for publication and will be formally accepted for publication once it meets all outstanding technical requirements.

Within one week, you’ll receive an e-mail detailing the required amendments. When these have been addressed, you’ll receive a formal acceptance letter and your manuscript will be scheduled for publication.

An invoice will be generated when your article is formally accepted. Please note, if your institution has a publishing partnership with PLOS and your article meets the relevant criteria, all or part of your publication costs will be covered. Please make sure your user information is up-to-date by logging into Editorial Manager at Editorial Manager® and clicking the ‘Update My Information' link at the top of the page. For questions related to billing, please contact billing support.

If your institution or institutions have a press office, please notify them about your upcoming paper to help maximize its impact. If they’ll be preparing press materials, please inform our press team as soon as possible -- no later than 48 hours after receiving the formal acceptance. Your manuscript will remain under strict press embargo until 2 pm Eastern Time on the date of publication. For more information, please contact onepress@plos.org.

Kind regards,

Mohamed Baklola

Academic Editor

PLOS One

Additional Editor Comments (optional):

The authors have adequately addressed all comments and concerns raised during the previous round of review.

The revised manuscript is substantially improved in terms of methodology, reporting, reproducibility, and presentation.

I have no further substantive comments or concerns.

I recommend the manuscript for acceptance and publication in its current form.

Reviewers' comments:

# Editor report: Acceptance letter

**DOI:** 10.1371/journal.pone.0357661.r006

**Authors:** Mohamed Baklola

PONE-D-25-28656R2

PLOS One

Dear Dr. Li,

I'm pleased to inform you that your manuscript has been deemed suitable for publication in PLOS One. Congratulations! Your manuscript is now being handed over to our production team.

At this stage, our production department will prepare your paper for publication. This includes ensuring the following:

* All references, tables, and figures are properly cited

* All relevant supporting information is included in the manuscript submission,

* There are no issues that prevent the paper from being properly typeset

You will receive further instructions from the production team, including instructions on how to review your proof when it is ready. Please keep in mind that we are working through a large volume of accepted articles, so please give us a few days to review your paper and let you know the next and final steps.

Lastly, if your institution or institutions have a press office, please let them know about your upcoming paper now to help maximize its impact. If they'll be preparing press materials, please inform our press team within the next 48 hours. Your manuscript will remain under strict press embargo until 2 pm Eastern Time on the date of publication. For more information, please contact onepress@plos.org.

You will receive an invoice from PLOS for your publication fee after your manuscript has reached the completed accept phase. If you receive an email requesting payment before acceptance or for any other service, this may be a phishing scheme. Learn how to identify phishing emails and protect your accounts at https://explore.plos.org/phishing.

If we can help with anything else, please email us at customercare@plos.org.

Thank you for submitting your work to PLOS One and supporting open access.

Kind regards,

PLOS One Editorial Office Staff

on behalf of

Dr. Mohamed Baklola

Academic Editor

PLOS One
